# Developing a Subset of ICNP^®^ Terminology for NICU and Neonatology Settings

**DOI:** 10.3390/healthcare14050594

**Published:** 2026-02-27

**Authors:** Valentina Tommasi, Laura E. A. Stabilini, Giulia Vercesi, Serena Rampini, Patrizio Sannino, Vincenza Aloia, Sara Marotta, Luca G. Re, Camilla Ripari, Stefania C. Rippa, Barbara Bassola, Maura Lusignani

**Affiliations:** 1 Niguarda Hospital, Ospedale Maggiore 3 Square, 20162 Milan, Italy; vincenza.aloia@ospedaleniguarda.it (V.A.); sara.marotta@ospedaleniguarda.it (S.M.); lucagiuseppe.re@ospedaleniguarda.it (L.G.R.); camilla.ripari@ospedaleniguarda.it (C.R.); stefaniaceleste.rippa@ospedaleniguarda.it (S.C.R.); barbara.bassola@ospedaleniguarda.it (B.B.); 2 Department of Biomedical Health Sciences, University of Milan, Carlo Pascal 36 Street, 20133 Milan, Italy; maura.lusignani@unimi.it; 3 Buzzi Children’s Hospital (ASST FBF-Sacco), Lodovico Castelvetro 32 Street, 20154 Milan, Italy; laura.stabilini@unimi.it; 4 Fondazione IRCCS Ca’ Granda Ospedale Maggiore Policlinico, Via Francesco Sforza 28, 20122 Milan, Italy; giulia.vercesi@policlinico.mi.it (G.V.); serena.rampini@policlinico.mi.it (S.R.); patrizio.sannino@policlinico.mi.it (P.S.)

**Keywords:** standardized nursing language, ICNP, subset, NICU, neonatology, electronic health record

## Abstract

**Highlights:**

**What are the main findings?**

- ICNP^®^ is a standardized nursing language that can represent nursing phenomena in NICU and neonatology settings; 576 terms make up a proposed Subset.
- SNOMED CT can represent the proposed ICNP^®^ Subset.

**What are the implications of the main findings?**

- The use of ICNP^®^ in NICU and neonatology settings could increase patient safety, nursing research, and the visibility of nursing care.
- The use of the proposed Subset may facilitate the introduction of EHRs into clinical practice in NICU and neonatology settings.

**Abstract:**

**Background/Objectives**: The International Classification for Nursing Practice (ICNP^®^) is a standardized nursing language that enables the description of nursing care through diagnoses, interventions, and outcomes. An ICNP^®^ Subset is a sub-group of ICNP^®^ terms appropriate for settings of practice, facilitating the direct use of the ICNP^®^ in nursing documentation. As far as we know, there are no Subsets concerning neonatology and Neonatal Intensive Care Unit (NICU) settings. The aim of this study is to develop a Subset of ICNP^®^ for NICU and neonatology settings, presenting terms that are validated and harmonized with SNOMED CT nomenclature. **Methods**: This is a two-phase study. In the first phase, ICNP^®^ terms were validated through a qualitative study using a four-round Delphi method and a focus group involving experts in NICU and neonatology settings and education. The second phase focused on harmonizing the proposed ICNP^®^ Subset with SNOMED CT. **Results**: A total of 479 ICNP^®^ terms belonging to the Diagnosis/Outcome (DC) and Intervention (IC) axes were validated by the experts. Of these, 99.65% were found to be compatible with SNOMED CT. In addition, 97 new terms (30 Diagnoses/Outcomes and 67 Interventions) were validated and are currently awaiting approval by the International Council of Nurses. Of the newly proposed terms, 93.81% were compatible with SNOMED CT. **Conclusions**: The proposed Subset consists of 576 ICNP^®^ terms, including 177 Diagnoses/Outcomes and 399 Interventions. Its implementation may support the adoption of electronic health records in neonatal and NICU settings and contribute to improving the quality and standardization of nursing care.

## 1. Introduction

Standardized nursing languages (SNLs) enable the structured and consistent description and documentation of nursing care across different clinical settings [1,2]. Their use has been associated with improvements in patient safety, the advancement of nursing research, and the articulation of nurses’ clinical judgement and decision-making processes [2,3,4,5,6,7,8]. Moreover, SNLs support the integration of electronic health records (EHRs) into clinical practice, enhancing the visibility, measurability, and comparability of nursing care, while ensuring a shared language that is essential for electronic information exchange [2,4,6,7,9,10,11,12].

The International Classification for Nursing Practice (ICNP^®^) is an agreed set of terms that can be used to record the observations and interventions of nurses across the world [13,14]. Developed by the International Council of Nurses (ICN), recognized by the World Health Organization (WHO), and currently available as a Reference Set (RefSet) within SNOMED CT, the ICNP^®^ represents a key instrument for semantic interoperability in clinical practice and electronic health documentation [4,15,16,17].

The 2019 release of the ICNP^®^ is available for consultation on a free browser provided by the ICN [14,18]. In the 2019 release, ICNP^®^ terms are organized into seven axes: Focus, Judgement, Client, Action, Means, Location, and Time [14]. These axes can be combined according to syntactic rules to construct nursing diagnoses, outcomes, and interventions [14]. In addition, two precoordinated axes, Diagnosis/Outcome (DC) and Intervention (IC), allow for the direct use of diagnoses and interventions without the need for syntactic construction. Within the ICNP^®^, the DC axis encompasses both nursing diagnoses and nursing outcomes [4,14]. The ICNP^®^ terms that have been included in the SNOMED CT nomenclature are DC and IC, the precoordinated terms. SNOMED CT is itself a combinatorial nomenclature, allowing diagnoses or interventions to be constructed using syntax. This possibility will enable the ICN to better define any syntax rules for nursing diagnoses and interventions.

To facilitate the implementation of the ICNP^®^ in clinical practice and to support the aggregation and analysis of nursing data, the ICN actively promotes the development of ICNP^®^ Subsets. These Subsets consist of selected nursing diagnoses, interventions, and outcomes tailored to specific areas of practice or patient populations [5,8,14,19,20,21]. Examples of ICNP^®^ Subsets or proposed ICNP^®^ Subsets are self-care in people with diabetes mellitus, fall prevention among the elderly in primary care, and care for breastfeeding women and children [5,8,19]. The use of Subsets in clinical practice can facilitate the development of appropriate setting-specific care plans, supporting nursing work and the safety of patients and their families [19].

Newborns represent a particularly vulnerable population characterized by multiple risk factors, especially in the case of preterm infants [7,22,23]. In this context, the adoption of EHRs has the potential to significantly enhance patient safety and quality of care [7,10,11,24]. Recent studies have highlighted the relevance of the ICNP^®^ in neonatal settings: an Italian study reported a high level of concordance between nursing documentation in NICUs and ICNP^®^ terms, suggesting the feasibility of developing a dedicated Subset, while a Brazilian study validated ICNP^®^ nursing diagnoses for preterm neonates admitted to NICUs through expert consensus [7,11].

However, to our knowledge, no studies presenting a comprehensive and validated ICNP^®^ Subset specifically designed for neonatology and NICU settings are available. The lack of standardized, selected, and validated nursing terms for a specific setting can slow down the use of EHRs in clinical practice, make it more difficult for students to learn contextual nursing care, and make it more difficult to search through data. Against this backdrop, the aim of the present study is to develop a Subset of the ICNP^®^ for NICU and neonatology settings, presenting terms that are validated and harmonized with SNOMED CT nomenclature.

## 2. Materials and Methods

This is a two-phase study.

### 2.1. First Phase: Validation of the ICNP^®^ Terms

The first phase focused on the validation of ICNP^®^ terms for the development of the Subset. A multicenter qualitative descriptive study was conducted using the Delphi method [8,21,25], consisting of a total of four rounds—the first two rounds to perform an initial selection of ICNP^®^ terms and the third and fourth rounds to assess the relevance and usefulness of the previously selected ICNP^®^ terms—complemented by a focus group [26,27,28,29]. The Delphi rounds and the focus group involved experts and nurses from two third-level NICUs of two big hospitals in Milan, from the Bachelor Nursing School and Bachelor Pediatric Nursing School of the University of Milan, and from the ICNP^®^ Italian Research and Development Centre. Across all phases, purposive non-probability sampling was adopted [30].

The first and second Delphi rounds, conducted in 2022, aimed to perform an initial selection of ICNP^®^ terms belonging to the Diagnosis/Outcome (DC) and Intervention (IC) axes. For this purpose, five experts were included, each with at least ten years of experience in NICUs and neonatology and with experience in university teaching or in the management of pediatric nursing care in neonatal settings. It was decided to ask five experts with extensive experience of clinical cases in these settings to carry out an initial selection that would not exclude terms capable of describing care in NICU and neonatology settings.

The third and fourth Delphi rounds, carried out in 2023 and 2024 respectively, aimed to assess the relevance and usefulness of the previously selected ICNP^®^ terms for NICU and neonatology settings. These rounds included nurses and pediatric nurses with at least two years of clinical experience in NICUs or neonatology: 40 participants in the third round and 32 in the fourth round. In both rounds, participants were also invited to suggest additional terms they considered to be missing from the description of nursing care in neonatal settings.

In February 2025, a focus group was conducted to validate the new terms proposed during the four Delphi rounds and to re-evaluate terms that had received comments regarding their clinical relevance but presented linguistic or translation issues. The focus group included eight experts: two NICU and neonatology experts with at least five years of experience; three nursing education experts who use the ICNP^®^ for teaching purposes, one of whom had clinical experience in NICUs; two pediatric nursing education experts who use the ICNP^®^ for teaching purposes, one of whom had clinical experience in NICUs; and one member of the Italian ICNP^®^ Research and Development Center.

After obtaining approval from the Ethics Committee, the participating hospitals, and the universities involved, all participants were informed in advance via email about the research project and the objectives of the phase in which they were participating. For each Delphi round, participants completed a structured form consisting of five columns: term identification number, ICNP^®^ code, axis of classification (DC or IC), ICNP^®^ term, and notes. Participants were asked to evaluate each ICNP^®^ DC and IC term using a four-point Likert scale based on its appropriateness for NICU and neonatology settings. The Item Content Validity Index (I-CVI) and the Item Content Validity Ratio (I-CVR) were calculated. According to Lawshe [31], the minimum acceptable I-CVR values vary depending on panel size: 0.99 for five experts, 0.75 for eight experts, 0.29 for forty experts, and 0.33 for thirty-two experts. The minimum acceptable I-CVI value was set at 0.80 [4,8,13,32,33,34]; terms with values between 0.70 and 0.79 were subjected to revision [34,35]. In the first and second Delphi rounds, given the early stage of Subset development and the planned continuation of the Delphi process, terms with I-CVI values between 0.70 and 0.79 were re-evaluated even when the I-CVR values were below the recommended threshold.

Notes and proposals for new terms collected during the third and fourth rounds were analyzed by the research group. With the support of the Italian ICNP^®^ Research and Development Center, new terms were constructed using ICNP^®^ terms from the seven axes, in accordance with ICNP^®^ syntactic rules.

For the focus group, participants received an email containing the list of terms already validated, which were not discussed during the session, and the terms to be examined, including newly proposed terms and those presenting linguistic or translation issues. The focus group was conducted via the Microsoft Teams^®^ platform; participants were sent the access link by e-mail. During the focus group, the aim and the rules of participation were explained. Each expert participated with their camera and audio active; the screen was shared, allowing the ICNP^®^ terms to be viewed for discussion regarding validation. Each term was presented and discussed until unanimous consensus was reached regarding its acceptance or exclusion; any modifications were made in real time and shared with all participants.

### 2.2. Second Phase: Harmonization with SNOMED CT

The second phase addressed the harmonization of the proposed ICNP^®^ Subset with the SNOMED CT. An analytical approach was adopted to systematically compare ICNP^®^ and SNOMED CT terms, in line with the existing literature on terminological harmonization [36,37,38,39,40]. Harmonization was performed using the SNOMED CT International Edition browser, the free ICNP^®^ browser (2019 version), and comparison tables based on the SNOMED CT–ICNP RefSet, provided by the ICNP^®^ Italian Research and Development Centre. As no Italian version of the SNOMED CT browser is currently available, the terms of the proposed Subset were compared with the English version of the ICNP^®^ browser using ICNP^®^ codes. The comparison tables provided by the ICNP^®^ Italian Research and Development Centre enabled 468 out of 479 terms to be matched. A total of 16 terms, 5 DC and 11 IC, and all newly proposed terms were reviewed by two members of the research group with a minimum English level of B2, one of whom was also part of the ICNP^®^ Italian Research and Development Centre. Both proposed their version of the SNOMED CT terms, then compared them, and disagreements were resolved by consensus. The research group then approved the proposed SNOMED CT terms. An equivalence table between ICNP^®^ and SNOMED CT was subsequently developed using Microsoft Word 365^®^.

### 2.3. Ethics

This study was conducted in agreement with the current privacy legislation and the principles stated in the Helsinki Declaration. The study was approved by the Ethics Committee of Niguarda Hospital on 16 February 2022 (approval number 86-16022022). The ICNP^®^ Italian Research and Development Centre was informed about the study and provided its approval. Written informed consent to participate in the study was obtained from all participants involved in the Delphi rounds and the focus group. Written consent for audio and video recording was additionally obtained from focus group participants.

## 3. Results

### 3.1. First Phase

#### 3.1.1. First and Second Delphi Round

Five experts (two men and three women; mean age 41.6 ± 8.47 years) participated, with an average of 15.4 ± 6.8 years of professional experience in NICUs or neonatology and 13.6 ± 7.1 years of experience in pediatric nursing education at the university level. Two experts had 10 and 13 years of experience in NICU management, and two had at least five years of ICNP^®^ use. During the first round, I-CVR and I-CVI analyses validated 497 ICNP^®^ terms (DC and IC axes) with values of 1.0. An additional 305 terms, with I-CVI values between 0.70 and 0.79 and I-CVR = 0.60, were re-evaluated in the second round, resulting in 250 terms being confirmed with I-CVI > 0.79. After the first and second rounds, a total of 747 ICNP^®^ terms (DC and IC) were selected. Experts also identified the need for new terms to better describe nursing care in NICU and neonatal settings.

#### 3.1.2. Third Delphi Round

Forty nurses (2 men and 38 women; mean age 34.97 ± 7.15 years; mean clinical experience 10.75 ± 6.71 years) evaluated the 747 previously selected terms. Nine participants reported having between 1 and 25 years of experience teaching pediatric nursing at university level or as clinical mentors on the ward. I-CVR and I-CVI analyses validated 409 terms, while 225 required revisions (I-CVI 0.70–0.79; I-CVR > 0.29), and 113 terms were removed.

#### 3.1.3. Fourth Delphi Round

Thirty-two nurses (31 women and 1 undisclosed; mean age 40.0 ± 10.6 years; mean experience 16.0 ± 11.3 years) evaluated the 225 terms from the third round. Twelve participants reported pediatric nursing teaching experience or experience as clinical mentors on the ward for between 1 and 22 years. I-CVR and I-CVI analyses validated 61 terms (I-CVR > 0.33; I-CVI > 0.79), while 9 IC terms with repeated translation discrepancies were suspended. Across the third and fourth rounds, experts proposed 173 new terms, which were reduced to 87 after review by the research group due to conceptual redundancy. After the fourth round, 470 ICNP^®^ terms (146 DC and 324 IC) were validated for the Subset. The 87 new terms suggested by participants (18 DC and 69 IC) underwent further evaluation in the focus group.

#### 3.1.4. Focus Group

Eight experts (seven women and one man; mean age 45.87 ± 10.62 years; mean experience 21.37 ± 11.08 years) participated. Two were pediatric nurses with more than five years of NICU experience; five were nursing educators using the ICNP^®^ in teaching, two of whom had NICU experience; and one was a member of the ICNP^®^ Italian Research and Development Centre. The session lasted 1 h 40 min.

During the focus group, the 87 new terms were individually discussed, and participants were asked to express their agreement or disagreement with the inclusion of the term in the proposed Subset or their considerations regarding the need for modification. Experts assessed lexical clarity, contextual appropriateness, and DC references for IC terms.

A total of 54 terms were accepted without modification, 21 were accepted after discussion and minor adjustments, 2 were excluded, and 10 were addressed for overlap with existing ICNP^®^ terms (five reassigned to existing terms, already validated in the Delphi rounds; five maintained with one newly proposed). Additionally, 17 new terms (4 IC and 13 DC) that were missing from the Subset and recognized as missing in relation to already validated DC or IC terms were proposed, discussed, and validated. Nine terms with translation disagreements were revised and accepted.

At the end of first phase, the proposed Subset comprised 479 ICNP^®^ terms from the 2019 release (147 DC and 332 IC) and 97 newly proposed terms (30 DC and 67 IC). Table 1 and Table 2 show examples of DC terms (Table 1) and IC terms (Table 2) present in the proposed Subset. The complete list of terms is available in the Supplementary Material.

### 3.2. Second Phase

#### Harmonization with SNOMED CT

Of the 479 validated ICNP^®^ terms, 463 (96.65%) were matched to SNOMED CT using the comparison tables provided by the ICNP^®^ Italian Research and Development Centre. Of the remaining 16 terms (3.33%), a search in the International SNOMED CT browser identified matches for 11 terms (4 DC and 7 IC), while 5 terms (1 DC and 4 IC) had no correspondence. The DC term without a match was “Improved electrolyte balance (10033518)”, and the four IC terms without a match were “Transporting patient (10020095)”, “Managing arterial blood flow (10050688)”, “Managing feeding device (10050769)”, and “Monitoring ascites (10051658)”. Thus, 475 (99.16%) validated terms out of 479 for the Subset found a possible match in SNOMED CT.

Among the 97 new ICNP^®^ terms, 91 (93.81%) had an exact or approximate SNOMED CT match. For the 30 DC terms, 26 (86.66%) matched SNOMED CT, and 4 (13.33%) had no match: “Effective Aspiration”, “Risk of Impaired Ventilatory Adaptation”, “Risk of Negative Response to Non-Pharmacological Pain Management”, and “Negative Response to Non-Pharmacological Pain Management”. Among the 67 IC terms, 65 (97.01%) matched SNOMED CT, while 2 (2.98%) had no correspondence: “Implementation of Non-Pharmacological Pain Management Techniques” and “Secretion Monitoring”.

## 4. Discussion

The aim of this study was to develop a Subset of the ICNP^®^ for NICU and neonatology settings, presenting terms that are validated and harmonized with SNOMED CT nomenclature. The result was a proposal for a solid base of terms that could be incorporated into the language, proposing an ICNP^®^ Subset that includes 479 terms from the 2019 release of ICNP^®^, with 147 belonging to the DC axis (Clinical Diagnosis) and 332 to the IC axis (Clinical Intervention). Additionally, 97 new terms were proposed (30 DC and 67 IC) pending final approval by the ICN.

### 4.1. First Phase: Validation of the ICNP^®^ Terms

Overall, nurses and experts recognized the ICNP^®^ terminology as appropriate, although some translation issues arose. These controversies led the experts in the focus group to revise certain terms, for example “Gastric tube care” or “Drainage tube care” or “Fracture cure”, where the problem concerned the translation of the term “cure”. These ambiguities were resolved by consulting the original English ICNP^®^ terms, which were often clearer than the Italian equivalents.

The study allowed the use of DC and IC axes already included in SNOMED CT and the creation of new terms using the seven-axis ICNP^®^ syntax. This approach ensured consistency with the official ICNP^®^ terminology and ISO standards, avoiding the need to standardize the proposed terms [25].

Since the ICNP^®^ terminology was not sufficiently complete for the neonatal context [7,11,14], nurses and experts were able to propose new terms to describe specific clinical phenomena [11,41]. The focus group enabled experts to evaluate the suggested terms in the Delphi rounds, ensuring that DC terms represented both diagnosis and outcome. When possible, related interventions were combined, for example: “Device Injury”, “No device injury”, and “Alternate the devices’s position”. This ability to combine terms facilitates clinical practice and paves the way for the use of artificial intelligence, such as Machine Learning and Deep Learning, in EHRs [42].

### 4.2. Second Phase: Harmonization with SNOMED CT

All terms in the proposed Subset were harmonized with SNOMED CT, the most comprehensive clinical terminology [7,16]. Collaboration with the ICNP^®^ Italian Research and Development Centre helped the research group obtain support regarding harmonization with SNOMED CT. A total of 96.65% of ICNP^®^ terms validated through the Delphi rounds found a match in SNOMED CT. Moreover, 86.4% of newly proposed terms were potentially harmonizable. Some non-harmonized terms describe specific nursing procedures, such as “Monitoring ascites” or “Managing feeding device” or “Managing arterial blood flow” or “Negative response to non-pharmacological pain management”. The ICNP^®^ syntax still allows for the combination of different terms to express complex concepts, supporting full integration with SNOMED CT [43]. Recent studies emphasize the importance of mapping the ICNP^®^ to SNOMED CT to facilitate the use of standardized nursing languages in EHRs [16,43,44]. The presence of the ICNP^®^ within SNOMED CT could facilitate the use of SNLs in EHRs [36].

### 4.3. Implications for Clinical Practice, Research, and Education

The proposed ICNP^®^ Subset introduced into clinical practice in an EHR system could allow for a targeted selection of terms describing nursing care, improving its usability for nurses. At the same time, its use in education could guide students in learning about the specific nursing care in NICU and neonatology settings. Previous research has investigated ICNP^®^ use in NICUs, particularly for preterm infants. Querido et al. [11] developed 145 ICNP^®^ nursing diagnoses for preterm infants; most corresponded to a DC term in our study, either exactly (“Bradycardia”) or similarly (“Hypotension” or “Hypertension,” corresponding to “Altered blood pressure” in ICNP^®^ and “Blood pressure alteration” in SNOMED CT). Another study [7] demonstrated that 288 ICNP^®^ terms describe nursing phenomena documented in clinical notes. All terms validated for the proposed Subset cover these phenomena. This further research provides an opportunity to explore in greater depth the possible use of the ICNP^®^ in clinical practice and education and also propose new terms that will then need to be analyzed and validated by the ICN.

The proposed ICNP^®^ Subset allows for the description of risks, problems, and nursing interventions for hospitalized neonates and their families in NICUs. Examples include nutritional risks, like “Interrupted breastfeeding” (10000774), “Difficulty performing breastfeeding” (10001098), “Effective breastfeeding” (10001411), “Managing enteral feeding” (10031776), “Teaching about feeding technique” (10045411), “Sucking reflex equivocal” (new term, 299758003—SNOMED CT, Finding), and “Excessive weight loss” (new term, 309257005—SNOMED CT, Finding). Other examples may refer to terms related to respiratory difficulties or infections or family support, like “Impaired breathing” (10001344), “Effective breathing” (10041334), “Implementing ventilatory weaning” (10050657), “Monitoring response to ventilatory weaning” (10051731), “Urinary tract infection” (10029915), “Risk for eye infection” (10032372), “Irrigation of nasal passages” (new term, 69378000—SNOMED CT, Procedure), “Family able to participate in care planning” (10035904), “Supporting family mourning process” (10026470), “Hospital admission, parent, for in-hospital child care” (new term, 51501005—SNOMED CT, Procedure). Previously undescribed phenomena [7,11], such as meconium or gastric residue issues, were validated by the focus group: “Meconium quantitation” (new term, 17695009—SNOMED CT, Procedure), “Monitoring procedure + Volume of drainage of gastric contents” (new term, 239516002 + 1162665001—SNOMED CT, Regime/Therapy + Observable entity).

### 4.4. Further Research Directions

In the future, further research may explore the actual use and usability of the proposed ICNP^®^ Subset. The proposed Subset represents an initial set of ICNP^®^ terms specific to NICU and neonatal settings. This proposed Subset can facilitate the adoption of EHRs, promote international comparisons, enhance the visibility of nursing work and reasoning, and support global research. The proposed new terms and the proposed Subset require international evaluation by the ICN and other ICNP^®^ experts.

### 4.5. Limitations

Study limitations include the small number and geographic concentration of participants, particularly from two big hospitals in a large city in northern Italy, the high workload during the Delphi rounds, and the lack of an official Italian translation of SNOMED CT, which may have led to search errors in the second phase.

## 5. Conclusions

This study aimed to develop an ICNP^®^ Subset for NICU and neonatology settings, presenting terms validated through SNOMED CT. The result was a proposal for a solid base of terms that could be become an ICNP^®^ Subset. The proposed ICNP^®^ Subset includes 147 Diagnoses/Outcomes, 332 Interventions, 30 newly proposed Diagnoses/Outcomes, and 67 newly proposed Interventions. Patients in NICU and neonatal settings, along with their families, often require complex, specialized nursing care. The proposed Subset attempts to provide standardized terminology that could support the use of SNLs to describe nursing care in these contexts. It could enable structured patient assessment, documentation, and measurement, facilitating the collection, interpretation, and recording of nursing data. This approach may enhance the visibility of nursing practice, support research, and promote the integration of nursing documentation into EHRs.

## Figures and Tables

**Table 1:** Examples of nursing diagnoses proposed in the Subset with a table showing the equivalence between ICNP^®^ terms included in the proposed Subset and SNOMED CT.

ICNP^®^ Code	Axis	Italian ICNP^®^ Browser	English ICNP^®^ Browser	SCTID	SNOMED CT Browser
10000567	DC	Stipsi	constipation	14760008	Constipation (finding)
10001098	DC	Difficoltà nell’allattamento al seno	difficulty performing breastfeeding	289084000	Difficulty performing breastfeeding (finding)
10001177	DC	Alterazione dello scambio gassoso	impaired gas exchange	70944005	Impaired gas exchange (finding)
10001385	DC	Stress dei genitori	parental stress	1236741000	Parenting stress (finding)
10027392	DC	Peso nei limiti della norma	weight within normal limits	43664005	Normal weight (finding)
10027964	DC	Efficace clearance delle vie aeree	effective airway clearance	1145305006	Able to clear airway effectively (finding)
10032372	DC	Rischio di infezione oculare	risk for eye infection	704360006	At increased risk for infection of eye (finding)
10035405	DC	Caregiver capace di prendersi cura in modo efficace	caregiver able to perform caretaking	1148835005	Caregiver able to perform caring activities (situation)
10039503	DC	Allattamento al seno esclusivo	exclusive breastfeeding	1145307003	Exclusively breastfed (finding)
10047245	DC	Minzione efficace	effective urination	102834005	Normal micturition (finding)

**Table 2:** Examples of nursing interventions proposed in the Subset with a table showing the equivalence between ICNP^®^ terms included in the proposed Subset and SNOMED CT.

ICNP^®^ Code	Axis	Italian ICNP^®^ Browser	English ICNP^®^ Browser	SCTID	SNOMED CT Browser
10006028	IC	Pianificare la dimissione con il caregiver familiare	discharge planning by family caregiver	710483006	Discharge planning by family caregiver (procedure)
10009872	IC	Attuare le linee guida sul dolore	implementing pain guideline	370823008	Implementation of pain guidelines (regime/therapy)
10012196	IC	Monitorare lo stato respiratorio	monitoring respiratory status	53617003	Monitoring of respiration (regime/therapy)
10024354	IC	Effettuare una dimostrazione sulla somministrazione della terapia	demonstrating medication administration	710574004	Demonstrating medication administration (procedure)
10032121	IC	Monitorare il peso	monitoring weight	307818003	Weight monitoring (regime/therapy)
10032703	IC	Screening dell’udito	screening hearing	710076008	Screening for hearing loss (procedure)
10038131	IC	Insegnare alla famiglia come eseguire l’igiene	teaching family about hygiene pattern	710753003	Family education about hygiene (situation)
10046052	IC	Raccogliere campioni di sangue arterioso	collecting arterial blood specimen	713143008	Collection of arterial blood specimen (procedure)
10051248	IC	Esaminare l’addome	examining abdomen	225162003	Examination of abdomen (procedure)
10051627	IC	Stimolare il neonato	stimulating newborn	1155834004	Stimulation of newborn (procedure)

## Data Availability

The original contributions presented in this study are included in the Supplementary Materials. Further inquiries can be directed to the corresponding author.

## Supplementary Materials

The following supporting information can be downloaded at: https://www.mdpi.com/article/10.3390/healthcare14050594/s1. Table S1: Equivalence table between ICNP^®^ terms included in the proposed Subset and SNOMED CT. Table S2: Equivalence table between newly proposed ICNP^®^ terms and SNOMED CT.

## Abbreviations

The following abbreviations are used in this manuscript:

DC axis	Diagnosis/Outcome Axis (Clinical Diagnosis)
EHR	Electronic Health Record
IC axis	Intervention Axis (Clinical Intervention)
ICNP	International Classification for Nursing Practice
NICU	Neonatal Intensive Care Unit
RefSet	Reference Set (SNOMED CT)
SNOMED CT	Systematized Nomenclature of Medicine Clinical Terms
SNL	Standardized Nursing Language
SCTID	SNOMED CT Identifier
