# Supplementary material for: Developing a Subset of ICNP® Terminology for NICU and Neonatology Settings
Source: Healthcare (Basel). 2026 Feb 27;14(5):594. doi: 10.3390/healthcare14050594 (PMC12984858; doi:10.3390/healthcare14050594)
Supplement: Supplementary file 1 [file healthcare-14-00594-s001.zip › Table S1.pdf]

Table S1. Equivalence table between ICNP® terms included in the proposed Subset and SNOMED CT (terms without a match are highlighted in yellow).

	ICNP® Code	Axis	Italian Browser ICNP®	English Browser ICNP®	SCTID	Browser SNOMED CT
1	10000454	DC	Dolore acuto	acute pain	274663001	Acute pain (finding)
2	10000567	DC	Stipsi	constipation	14760008	Constipation (finding)
3	10000630	DC	Diarrea	diarrhoea	62315008	Diarrhea (finding)
4	10000757	DC	Ipertermia	hyperthermia	50177009	Body temperature above reference range (finding)
5	10000761	DC	Ipotermia	hypothermia	386689009	Hypothermia (finding)
6	10000774	DC	Interruzione dell'allattamento al seno	interrupted breastfeeding	1149253002	Breastfeeding temporarily discontinued (finding)
7	10001080	DC	Alterata integrità dei tessuti	impaired tissue integrity	48851009	Impaired tissue integrity (finding)
8	10001098	DC	Difficoltà nell'allattamento al seno	difficulty performing breastfeeding	289084000	Difficulty performing breastfeeding (finding)
9	10001177	DC	Alterazione dello scambio gassoso	impaired gas exchange	70944005	Impaired gas exchange (finding)
10	10001290	DC	Alterazione dell'integrità cutanea	impaired skin integrity	7919002	Impaired skin integrity (finding)
11	10001316	DC	Alterazione della respirazione	impaired breathing	129895003	Impaired spontaneous ventilation (finding)
12	10001344	DC	Perfusione tissutale inefficace	ineffective tissue perfusion	409054008<br><br>409055009	Alteration in tissue perfusion (finding)<br><br>Ineffective tissue perfusion (finding)
13	10001359	DC	Alterata funzionalità del sistema urinario	impaired urinary system function	1156448003	Impaired urinary system function (finding)
14	10001385	DC	Stress dei genitori	parental stress	1236741000	Parenting stress (finding) en Parental stress
15	10001411	DC	Efficace allattamento al seno	effective breastfeeding	69840006	Normal breastfeeding (finding) Effective breastfeeding
16	10015053	DC	Rischio di stipsi	risk for constipation	129691005	At increased risk for constipation (finding) en At risk for constipation
17	10015122	DC	Rischio di caduta	risk for fall	129839007	At increased risk for falls (finding) en At risk for falls
18	10015133	DC	Rischio di infezione	risk for infection	78648007	At increased risk for infection (finding) en At risk for infections
19	10015146	DC	Rischio di lesione	risk for injury	81763001	At increased risk for traumatic injury (finding) en Injury risk
20	10015237	DC	Rischio di alterazione dell'integrità cutanea	risk for impaired skin integrity	70693003	At increased risk for impaired skin integrity (finding) en At risk for impaired skin integrity

21	10015263	DC	Rischio di lesione da posizionamento perioperatorio	risk for perioperative positioning injury	129703005	At increased risk for perioperative positioning injury (finding)<br>en At risk for perioperative positioning injury
22	10015339	DC	Rischio di morte infantile improvvisa	risk for sudden infant death	392562007	At increased risk for sudden infant death syndrome (finding)<br>en At risk for sudden infant death syndrome
23	10017268	DC	Rischio di emorragia	risk for haemorrhaging	04348005	At increased risk of hemorrhage (finding)<br>en At risk of hemorrhage
24	10021742	DC	Stress eccessivo, sovraccarico di stress	stress overload	424582000	Stress overload (finding)
25	10021885	DC	Occlusione intestinale	faecal impaction	44635007	Fecal impaction (disorder)
26	10022473	DC	Mancanza di sostegno familiare	lack of family support	704489009	Lack of family support (finding)
27	10022626	DC	Effetti collaterali della terapia farmacologica	medication side effect	401207004	Medication side effects present (finding)<br>en Medication side-effect
28	10022846	DC	Tremore	tremor	26079004	Tremor (finding)
29	10022931	DC	Alterata funzionalità dell'apparato gastrointestina-le	impaired gastrointestinal system function	61578001	Abnormal digestive tract function (finding)
30	10022949	DC	Alterazione del sistema cardiovascolare	impaired cardiovascular system	129898001	Cardiovascular system alteration (finding)
31	10022954	DC	Pressione arteriosa alterata	altered blood pressure	129899009	Blood pressure alteration (finding)
32	10023009	DC	Alterato apporto di nutrienti	impaired nutritional intake	445261000<br>124106	Nutrition impaired (finding)
33	10023013	DC	Rischio di deficit nutrizionale	risk for impaired nutritional intake	129845004	At increased risk for imbalanced nutrition, less than body requirements (finding)<br>en At increased risk of nutritional deficit
34	10023032	DC	Infezione	infection	40733004	Infectious disease (disorder)<br>Infection
35	10023130	DC	Dolore	pain	22253000	Pain (finding)
36	10023148	DC	Ferita chirurgica	surgical wound	419635000	Surgical wound finding (finding)
37	10023362	DC	Alterata funzionalità del sistema respiratorio	impaired respiratory system function	129893005	Respiratory alteration (finding)
38	10024930	DC	Sonno adeguato	adequate sleep	113743700<br>5	Has adequate sleep (finding)
39	10025002	DC	Adeguato stato nutrizionale	positive nutritional status	248324001	Well nourished (finding)
40	10025588	DC	Sofferenza	suffering	706873003	Suffering (finding)
41	10025590	DC	Privacy	privacy	397644002	Right to privacy maintained (finding)

42	10025638	DC	Atteggiamento positivo del caregiver	positive caregiver attitude	1149083004	Caregiver has positive attitude (situation)
43	10025705	DC	Agitazione	agitation	24199005	Feeling agitated (finding)
44	10025722	DC	Irrequietezza, inquietudine	restlessness	162221009	Restlessness (finding)
45	10025798	DC	Ulcere da pressione	pressure ulcer	399912005	Pressure ulcer (disorder)
46	10025820	DC	Controllo dei sintomi	symptom control	1149243003	Managing to control symptoms (finding)
47	10025831	DC	Controllo del dolore	pain control	1156446004	Demonstrates adequate pain control (finding)
48	10025981	DC	Vomito	vomiting	422400008	Vomiting (disorder)
49	10027177	DC	Inalazione accidentale, involontaria	aspiration	413585005	Aspiration into respiratory tract (finding)
50	10027226	DC	Alterazione del sonno	impaired sleep	26677001	Sleep pattern disturbance (finding)
51	10027274	DC	Bradicardia	bradycardia	48867003	Bradycardia (finding)
52	10027288	DC	Tachicardia	tachycardia	3424008	Tachycardia (finding)
53	10027316	DC	Sottopeso	underweight	248342006	Underweight (finding)
54	10027328	DC	Rischio di ipertermia	risk for hyperthermia	704362003	At increased risk of hyperthermia (finding) en At risk of hyperthermia
55	10027337	DC	Rischio di lesione da pressione	risk for pressure ulcer	285304000	At risk of pressure injury (finding)
56	10027392	DC	Peso nei limiti della norma	weight within normal limits	43664005	Normal weight (finding)
57	10027482	DC	Edema periferico	peripheral oedema	271809000	Peripheral edema (disorder)
58	10027550	DC	Iperglicemia	hyperglycaemia	80394007	Hyperglycemia (disorder)
59	10027566	DC	Ipoglicemia	hypoglycaemia	302866003	Hypoglycemia (disorder)
60	10027647	DC	Pressione arteriosa nei limiti della norma	blood pressure within normal limits	2004005	Normal blood pressure (finding)
61	10027652	DC	Temperatura corporea nei limiti della norma	body temperature within normal limits	87273009	Temperature normal (finding)
62	10027773	DC	Stress del caregiver	caregiver stress	129891007	Caregiver role strain (finding)
63	10027917	DC	Riduzione del dolore	reduced pain	816133002	Sensation of pain reduced (finding)
64	10028160	DC	Adeguata funzionalità del sistema respiratorio	effective respiratory system function	22803001	Normal respiratory function (finding)
65	10027964	DC	Efficace clearance delle vie aeree	Effective Airway Clearance	1145305006	Able to clear airway effectively (finding)
66	10028488	DC	Integrità della mucosa del cavo orale	tissue integrity of oral mucous membrane	1149248007	Intact oral mucosa (finding)
67	10028517	DC	Miglioramento dell'integrità cutanea	improved skin integrity	1144884009	Improvement in level of integrity of skin (finding)
68	10028555	DC	Integrità tissutale	tissue integrity	225547008	Finding of integrity of skin (finding)
69	10028670	DC	Risposta efficace alla terapia farmacologica	effective response to medication	1137679005	Good response to medication (finding)
70	10028806	DC	Assenza di emorragia	no bleeding	1141705002	No bleeding (situation)

71	10028945	DC	Assenza di infezione	no infection	397680002	Absence of signs and symptoms of infection (situation)
72	10028966	DC	Assenza di lesione	no injury	224964008	No injuries apparent (finding)
73	10029008	DC	Assenza di dolore	no pain	81765008	No pain (situation)
74	10029065	DC	Assenza di lesione da pressione	no pressure ulcer	115702700 4	No pressure injury (situation)
75	10029181	DC	Assenza di vomito	no vomiting	162062008	No vomiting (situation)
76	10029229	DC	Frequenza cardiaca nei limiti della norma	heart rate within normal limits	76863003	Normal heart rate (finding)
77	10029264	DC	Assenza di dispnea	no dyspnoea	161938003	No breathlessness (situation)
78	10029405	DC	Caduta	fall	161898004	Falls (finding)
79	10029728	DC	Ingorgo mammario	breast engorgement	237355007	Milk engorgement of breast (disorder)
80	10029737	DC	Ustione	burn wound	125666000	Burn (disorder)
81	10029856	DC	Problema di sicurezza ambientale	environmental safety problem	704300009	Environmental safety problem (finding)
82	10029873	DC	Alterato apporto di liquidi	impaired fluid intake	249481003	Finding of insufficient fluid intake (finding)
83	10029915	DC	Infezione del tratto urinario	urinary tract infection	68566005	Urinary tract infectious disease (disorder)
84	10029927	DC	Infiammazione	inflammation	257552002	Inflammation (qualifier value)
85	10029936	DC	Lesione	injury	417163006	Traumatic or non-traumatic injury (disorder)<br>en Injury
86	10029958	DC	Carenza di conoscenze sull'allattamento al seno	lack of knowledge of breast feeding	704375003	Deficient knowledge of breast feeding (finding)
87	10030003	DC	Edema linfatico	lymphatic oedema	234097001	Lymphedema (disorder)
88	10030116	DC	Edema declive	weeping oedema	443903000	Extravasation of lymph onto skin (disorder)
89	10032372	DC	Rischio di infezione oculare	risk for eye infection	704360006	At increased risk for infection of eye (finding)<br>en At risk for infection of eye (finding)
90	10033502	DC	Miglioramento dell'equilibrio acido-base	improved acid base balance	114485700 8	Improvement in acid-base balance (finding)
91	10033518	DC	Miglioramento dell'equilibrio elettrolitico	improved electrolyte balance	.	
92	10033539	DC	Squilibrio acido-base	acid base imbalance	704429007	Abnormal acid-base balance (finding)
93	10033541	DC	Squilibrio elettrolitico	electrolyte imbalance	105593004	Electrolyte imbalance (disorder)
94	10033560	DC	Alterazione della termoregola-zione	impaired thermoregulation	85623003	Ineffective thermoregulation (finding)
95	10033685	DC	Livello glicemico nei limiti della norma	blood glucose within normal limits	166921001	Blood glucose normal (finding)

96	10033721	DC	Bilancio idrico nei limiti della norma	fluid balance within normal limits	1156884002	Fluid balance consistent with requirement (finding)
97	10033848	DC	Termoregola- zione efficace	effective thermoregula-tion	1144584004	Normal thermoregulation (finding)
98	10034838	DC	Coping del caregiver efficace	effective caregiver coping	425578005	Caregiver able to cope (finding)
99	10034990	DC	Iperbilirubine-mia	Hyperbilirubi= naemia	14783006	Hyperbilirubinemia (disorder)
100	10035020	DC	Apnea	apnoea	1023001	Apnea (finding)
101	10035031	DC	Rischio di apnea	risk for apnoea	704422003	At increased risk of apnea (finding)<br>en At risk of apnea
102	10035054	DC	Bilirubinemia nei limiti della norma	serum bilirubin within normal limits	166611006	Serum bilirubin normal (finding)
103	10035405	DC	Caregiver capace di prendersi cura in modo efficace	caregiver able to perform caretaking	1148835005	Caregiver able to perform caring activities (situation)
104	10035414	DC	Incapacità del caregiver di prendersi cura in modo efficace	impaired ability of caregiver to perform caretaking	423754004	Difficulty with caretaking responsibilities (finding)
105	10035569	DC	Miglioramento dello stato nutrizionale	improved nutritional status	1145522009	Improvement in nutritional status (finding)
106	10035666	DC	Essere pronti per la dimissione	readiness for discharge	430567009	Ready for discharge (finding)
107	10035904	DC	Famiglia capace di partecipare al piano di cura	family able to participate in care planning	1148840002	Family able to participate in care planning (situation)
108	10036370	DC	Tolleranza alla dieta	diet tolerance	1144522003	Tolerating diet (finding)
109	10037305	DC	Funzionalità cardiaca alterata	impaired cardiac function	105981003	Disorder of cardiac function (disorder)
110	10037604	DC	Rischio di effetti collaterali della terapia farmacologica	risk for medication side effect	704417003	At risk of medication side effect (finding)
111	10037615	DC	Rischio di compromissione del sonno	risk for impaired sleep	704414005	At risk of sleep impairment (finding)
112	10037658	DC	Conoscenza della malattia da parte della famiglia	family knowledge of disease	1144471005	Family demonstrates knowledge of disease process (situation)
113	10039503	DC	Allattamento al seno esclusivo	exclusive breastfeeding	1145307003	Exclusively breastfed (finding)
114	10039910	DC	Inadeguato controllo del dolore	inadequate pain control	704675005	Inadequate pain control (finding)
115	10040085	DC	Controllo dei sintomi della sindrome da astinenza	withdrawal symptom control	720822008	Withdrawal symptom (finding)
116	10040160	DC	Sedato	sedated	17971005	Sedated (finding)
117	10040457	DC	Rischio di complicanze della stomia	risk for stoma complications	704307007	At risk of complication of stoma (finding)
118	10040765	DC	Crisi di astinenza	withdrawn behaviour	276249004	Withdrawn behavior (finding)
119	10040875	DC	Qualità di vita	quality of life	1156447008	Good quality of life (finding)
120	10041334	DC	Efficace respirazione	effective breathing	288848001	Able to breathe (finding)

121	10041539	DC	Febbre	fever	386661006	Fever (finding)
122	10041587	DC	Rischio di vomito	risk for vomiting	704413004	At increased risk of vomiting (finding)<br>en At risk of vomiting
123	10041807	DC	Rischio di infezioni crociate	risk for cross infection	225943002	At increased risk of cross-infection (finding)<br>en At risk of cross-infection
124	10041882	DC	Disidratazione	dehydration	34095006	Dehydration (disorder)
125	10041895	DC	Rischio di disidratazione	risk for dehydration	704420006	At increased risk of dehydration (finding)<br>en At risk of dehydration
126	10042031	DC	Rischio di ipervolemia	risk for hypervolaemia	129849005	At increased risk for excess fluid volume (finding)<br>en At risk for excess fluid volume
127	10042049	DC	Rischio di ipovolemia	risk for hypovolaemia	50048006	At increased risk for deficient fluid volume (finding)<br>en At risk for deficient fluid volume
128	10042065	DC	Adeguata idratazione	adequate hydration	1144674006	Adequately hydrated (finding)
129	10042335	DC	Squilibrio idrico	fluid imbalance	190902006	Fluid imbalance (disorder)
130	10042390	DC	Assenza di complicanze della stomia	no stoma complication	1157057008	No complication of stoma (situation)
131	10042728	DC	Interazioni farmacologiche avverse	adverse medication interaction	448177004	Adverse drug interaction (disorder)
132	10043953	DC	Dolore addominale	abdominal pain	21522001	Abdominal pain (finding)
133	10043982	DC	Proteinuria	proteinuria	29738008	Proteinuria (finding)
134	10044239	DC	Alterazione della perfusione tissutale periferica	impaired peripheral tissue perfusion	704449003	Impairment of peripheral tissue perfusion (finding)
135	10045668	DC	Convulsioni	seizure	91175000	Seizure (finding)
136	10046676	DC	Tensione mammaria	breast tenderness	55222007	Breast tenderness (finding)
137	10047060	DC	Escoriazione	excoriation	247444006	Excoriation of skin (disorder)
138	10047073	DC	Secchezza cutanea	dry skin	16386004	Dry skin (finding)
139	10047136	DC	Alterazione della funzionalità del sistema circolatorio	impaired circulatory system function	733741005	Impaired cardiovascular system function (finding)
140	10047143	DC	Tosse	cough	49727002	Cough (finding)
141	10047245	DC	Minzione efficace	effective urination	102834005	Normal micturition (finding)
142	10047311	DC	Assenza di convulsioni	no seizure	370994008	Seizure free (finding)
143	10050516	DC	Alterazione dei parametri vitali	altered vital sign	41236000	Abnormal vital signs (finding)
144	10051932	DC	Rischio di embolia	risk for embolism	1144780006	At increased risk of embolism (finding)

145	10051950	DC	Rischio di infezione urinaria	risk for urinary infection	225956009	At increased risk of urinary tract infection (finding)<br>en At risk of urinary tract infection
146	10052082	DC	Motilità efficace	effective active range of motion	298229008	Active range of joint movement normal (finding)
147	10052095	DC	Motilità alterata	impaired active range of motion	298222004	Active range of joint movement reduced (finding)
1	10001804	IC	Somministrare farmaci e soluzioni	administering medication and solution	370773004	Administration of prescribed medications and solutions (procedure)
2	10001827	IC	Somministrazione di terapia profilattica	administering prophylactic treatment	710170000	Administration of prophylactic treatment (procedure)
3	10001938	IC	Sostenere, tutelare il paziente	advocating for patient	370770001	Acting as individual patient advocate (regime/therapy)
4	10002706	IC	Valutare lo stato cardiaco attraverso dispositivi di monitoraggio	assessing cardiac status using monitoring device	710839006	Assessment of cardiac status using monitoring device (procedure)
5	10002710	IC	Valutare il controllo del dolore	assessing control of pain	370778008	Assessment of pain control (procedure)
6	10002809	IC	Valutare il rischio di ipotermia	assessing risk for hypothermia	372033006	Hypothermia risk assessment (procedure)
7	10004073	IC	Classificare le ferite chirurgiche	categorising surgical wound	710504000	Categorizing surgical wound (procedure)
8	10004588	IC	Raccogliere campioni	collecting specimen	17636008	17636008 Specimen collection (procedure)
9	10005093	IC	Sorveglianza continua	continuous surveillance	372031008	Maintains continuous surveillance (regime/therapy)
10	10006016	IC	Pianificare la dimissione	discharge planning	371754007	Discharge planning (procedure)
11	10006028	IC	Pianificare la dimissione con il caregiver familiare	discharge planning by family caregiver	710483006	Discharge planning by family caregiver (procedure)
12	10006966	IC	Assicurare la continuità assistenziale	ensuring continuity of care	115670500<br>8	Ensuring continuity of care (procedure)
13	10007169	IC	Valutare la funzione respiratoria dopo l'operazione	evaluating respiratory status after operation	49730009	Post-operative follow-up, anesthesia (procedure)
14	10007182	IC	Valutare la risposta ai farmaci	evaluating response to medication	370807008	Evaluation of response to medications (procedure)
15	10007202	IC	Valutare la perfusione tissutale dopo l'operazione	evaluating tissue perfusion after operation	370802002	Evaluation of postoperative tissue perfusion (procedure)
16	10007218	IC	Valutare la guarigione della lesione	evaluating wound healing	372032001	Evaluation of wound healing progress (procedure)

17	10009683	IC	Identificare gli ostacoli alla comunicazione	identifying obstruction to communication	372035004	Identification of barriers to communication (regime/therapy)
18	10009696	IC	Identificare il rischio di emorragia	identifying risk for haemorrhaging	710770006	Identification of risk of hemorrhage (procedure)
19	10009872	IC	Attuare le linee guida sul dolore	implementing pain guideline	370823008	Implementation of pain guidelines (regime/therapy)
20	10010503	IC	Interpretare i risultati dell'emogas= analisi arteriosa	interpreting arterial blood gas result	370850003	Evaluation of arterial blood gas studies (procedure)
21	10011527	IC	Mantenere la dignità e la privacy	maintaining dignity and privacy	372061000	Maintaining the patient's dignity and privacy (procedure)
22	10011639	IC	Gestire la raccolta dei campioni	managing specimen collection	410359006	Specimen collection case management (procedure)
23	10011641	IC	Gestire i farmaci	managing medication	182832007	Procedure related to management of drug administration (procedure)
24	10011656	IC	Gestire il trattamento dei campioni	managing management of specimen	385832000	Specimen care management (procedure)
25	10011660	IC	Gestire il dolore	managing pain	278414003	Pain management (procedure)
26	10011694	IC	Gestire il trasporto	managing transporting	410365006	Transportation case management (procedure)
27	10012165	IC	Monitorare la temperatura corporea	monitoring body temperature	133879001	Monitoring of patient temperature (regime/therapy)
28	10012196	IC	Monitorare lo stato respiratorio	monitoring respiratory status	53617003	Monitoring of respiration (regime/therapy)
29	10012203	IC	Monitorare segni e sintomi di infezione	monitoring signs and symptoms of infection	370835007	Monitoring for signs and symptoms of infection (regime/therapy)
30	10014761	IC	Posizionare un paziente	positioning patient	229824005	Positioning patient (procedure)
31	10015484	IC	Disinfettare la cute prima di una operazione	preparing skin before operation	28792004	Preoperative preparation of skin (procedure)
32	10015631	IC	Prevenire lesioni da agenti chimici	preventing chemical injury	370827009	Implementation of protective measures to prevent skin and tissue injury
33	10015649	IC	Prevenire le infezioni crociate	preventing cross infection	710980000	Prevention of cross infection (procedure)
34	10015683	IC	Prevenire le lesioni da agenti meccanici	preventing mechanical injury	370829007	Implementation of protective measures to prevent skin or tissue injury due
35	10015704	IC	Prevenire le lesioni da agenti termici	preventing thermal injury	370828004	Implementation of protective measures to prevent skin or tissue injury due
36	10015817	IC	Promuovere una adeguata termoregolazio-ne	promoting positive thermoregula- tion	180333002	Body temperature modification and control (procedure)

37	10015919	IC	Tutela dei diritti del paziente	protecting patient right	386386008	Patient rights protection (procedure)
38	10016168	IC	Venipuntura	venipuncture	22778000	Venipuncture (procedure)
39	10020095	IC	Trasportare pazienti	transporting patient		
40	10020743	IC	Verificare la presenza del consenso informato prima dell'intervento	verifying consent before operation	370861006	Verification of consent for planned procedure (procedure)
41	10020758	IC	Verificare l'identità del paziente prima dell'intervento	verifying patient before operation	370786008	Confirming patient identity before operative/invasive procedure
42	10020770	IC	Verificare il sito e il lato dell'intervento chirurgico	verifying surgical act site and laterality	409056005	Verification of surgical site and laterality (procedure)
43	10021695	IC	Effettuare una dimostrazione sulla tecnica di iniezione sottocutanea	demonstrating subcutaneous injection technique	710573005	Demonstration of subcutaneous injection technique (procedure)
44	10021719	IC	Educare la famiglia riguardo alla malattia	teaching family about disease	710755005	Family education about disease (situation)
45	10021837	IC	Gestire gli effetti collaterali dei farmaci	managing medication side effect	396081009	Adverse drug reaction prevention management (procedure)
46	10023084	IC	Somministrare la terapia del dolore	administering pain medication	52685006	Administration of analgesic (procedure)
47	10023520	IC	Valutare il rischio di cadute	assessing risk for falls	414191008	Fall risk assessment (procedure)
48	10023565	IC	Collaborare con il medico	collaborating with physician	709755006	Liaising with physician (procedure)
49	10023577	IC	Collaborare con l'assistente sociale	collaborating with social worker	709753004	Liaising with social worker (procedure)
50	10023888	IC	Gestire il programma farmacologico	managing medication regime	115669700 2	Coordination of medication regime (regime/therapy)
51	10024171	IC	Predisporre il servizio di trasporto	arranging transportation service	428632005	Transportation request (procedure)
52	10024331	IC	Chiedere un consulto per la gestione del dolore	consulting for pain management	420650002	Consultation for pain (procedure)
53	10024354	IC	Effettuare una dimostrazione sulla somministrazione della terapia	demonstrating medication administration	710574004	Demonstrating medication administration (procedure)
54	10024493	IC	Fornire materiale educativo	providing instructional material	445283009	Provision of educational material (procedure)
55	10024570	IC	Sostenere il caregiver	supporting caregiver	386229000	Caregiver support (regime/therapy)
56	10024589	IC	Sostenere il processo decisionale	supporting decision making process	133920001	Decision making encouragement (procedure)
57	10025444	IC	Somministrazione dei farmaci	administering medication	18629005	Administration of drug or medicament (procedure)
58	10026119	IC	Valutare il dolore	assessing pain	225399009	Pain assessment (procedure)

59	10026399	IC	Garantire la privacy	providing privacy	710920002	Provision of privacy (procedure)
60	10026462	IC	Sostenere il processo decisionale nel contesto familiare	supporting family decision making process	710867002	Support for family decision making process (regime/therapy)
61	10026470	IC	Sostenere la famiglia nell'elaborazione del lutto	supporting family mourning process	710865005	Support for family mourning process (regime/therapy)
62	10026489	IC	Sostenere l'elaborazione del lutto	supporting mourning process	395076009	Bereavement support (regime/therapy)<br>en Support mourning process
63	10030383	IC	Somministrare antibiotici	administering antibiotic	68322007	Administration of antibiotic (procedure)
64	10030417	IC	Somministrare l'insulina	administering insulin	39543009	Administration of insulin (procedure)
65	10030429	IC	Somministrare i vaccini	administering vaccine	33879002	Administration of vaccine to produce active immunity (procedure)
66	10030438	IC	Somministrare vitamina B12	administering vitamin B12	709544008	Administration of vitamin B12 (procedure)
67	10030493	IC	Predisporre il trasporto dei dispositivi	arranging transport of device	710951009	Arranging transportation of device (procedure)
68	10030570	IC	Valutare lo sviluppo del bambino	assessing child development	408984009	Growth and development care assessment (procedure)
69	10030591	IC	Valutare la conoscenza della malattia da parte della famiglia	assessing family knowledge of disease	709473008	Assessment of family knowledge of disease (situation)
70	10030660	IC	Valutare lo stato nutrizionale	assessing nutritional status	1759002	Assessment of nutritional status (procedure)
71	10030687	IC	Valutare al momento del ricovero	admission assessment	406152008	Admission assessment (procedure)
72	10030710	IC	Valutare il rischio di lesioni da pressione	assessing risk for pressure ulcer	225392000	Pressure ulcer risk assessment (procedure)
73	10030775	IC	Valutare lo stato di perfusione tissutale	assessing tissue perfusion	711014008	Assessment of tissue perfusion (procedure)
74	10030799	IC	Valutare la lesione	assessing wound	225395003	Wound assessment (procedure)
75	10030884	IC	Eseguire cateterismo vescicale	catheterising urinary bladder	410024004	Catheterization of urinary bladder (procedure)
76	10030911	IC	Controllare l'identità del paziente	checking patient identity	710958003	Checking patient identity (procedure)
77	10030924	IC	Controllare la sicurezza dei dispositivi	checking device safety	710956004	Checking device safety (procedure)
78	10031140	IC	Eseguire un test diagnostico	diagnostic testing	103693007	Diagnostic procedure (procedure)
79	10031164	IC	Vestire il paziente	dressing patient	313332003	Dressing patient (procedure)
80	10031252	IC	Valutare il piano di cura	evaluating care plan	712744002	Evaluation of care plan (procedure)
81	10031275	IC	Eseguire la cura oculare	eye care	225363004	Eye care (regime/therapy)

82	10031592	IC	Cura del sito di inserzione di un dispositivo invasivo	invasive device site care	370771002	Maintenance of invasive device (procedure)
83	10031724	IC	Gestire una linea di infusione centrale	managing central line	722440006	Management of central venous catheter (procedure)
84	10031776	IC	Gestire i dispositivi	managing device	363108004	Equipment-related management procedure (procedure)
85	10031795	IC	Gestire l'alimentazione enterale	managing enteral feeding	408909002	Enteral feeding management (procedure)
86	10031880	IC	Gestire la nefrostomia	managing nephrostomy care	710064008	Management of nephrostomy care (procedure)
87	10031908	IC	Gestire l'alimentazione parenterale	managing parenteral feeding	408914003	Parenteral feeding management (procedure)
88	10031954	IC	Gestire la cura delle stomie	managing stoma care regime	710078009	Management of stoma care (procedure)
89	10031965	IC	Gestire i sintomi	managing symptom	713148004	Symptom management (procedure)
90	10031977	IC	Gestire il catetere urinario	managing urinary catheter	410253009	Urinary catheter care management (procedure)
91	10031996	IC	Misurare la pressione arteriosa	measuring blood pressure	46973005	Blood pressure taking (procedure)
92	10032006	IC	Misurare la temperatura corporea	measuring body temperature	56342008	Temperature taking (procedure)
93	10032034	IC	Monitorare la glicemia	monitoring blood glucose	698472009	Blood glucose monitoring (regime/therapy)
94	10032047	IC	Monitorare la saturazione di ossigeno mediante l'uso di pulsossimetro	monitoring blood oxygen saturation using pulse oximeter	284034009	Pulse oximetry monitoring (regime/therapy)
95	10032052	IC	Monitorare la pressione arteriosa	monitoring blood pressure	135840009	Blood pressure monitoring (regime/therapy)
96	10032099	IC	Monitorare i risultati degli esami di laboratorio	monitoring laboratory result	410394004	Lab findings surveillance (regime/therapy)
97	10032113	IC	Monitorare i parametri vitali	monitoring vital signs	304495004	Monitoring of blood pressure, temperature, pulse rate and respiratory rate
98	10032121	IC	Monitorare il peso	monitoring weight	307818003	Weight monitoring (regime/therapy)
99	10032184	IC	Eseguire l'igiene orale	oral care	717778001	Mouth care (regime/therapy)
100	10032420	IC	Trattamento delle ulcere da pressione	pressure ulcer care	225357008	Pressure ulcer care (regime/therapy)
101	10032477	IC	Promuovere l'igiene	promoting hygiene	710150006	Promotion of hygiene (procedure)
102	10032630	IC	Rimuovere i punti di sutura	removing suture	30549001	Removal of suture (procedure)
103	10032703	IC	Screening dell'udito	screening hearing	710076008	Screening for hearing loss (procedure)
104	10032719	IC	Screening dello sviluppo infantile	screening infant development	15376006	Infant development screening (procedure)

105	10032735	IC	Screening della vista	screening vision	408760009	Vision screening (procedure)
106	10032757	IC	Cura della cute	skin care	225360001	Skin care (regime/therapy)
107	10032788	IC	Cura delle stomie	stoma care	225194008	Stoma care procedure (regime/therapy)
108	10032816	IC	Sostenere l'allattamento al seno	supporting breastfeeding	408883002	Breastfeeding support (regime/therapy)
109	10032844	IC	Sostenere la famiglia	supporting family	243115004	Family support (regime/therapy)
110	10032863	IC	Cura della ferita chirurgica	surgical wound care	226007004	Post-surgical wound care (regime/therapy)
111	10032994	IC	Educare riguardo un'efficace relazione genitore figlio	teaching about effective parenting	414384004	Parenting education (procedure)
112	10033001	IC	Insegnare il controllo del peso	teaching about effective weight	410200000	Weight control education (procedure)
113	10033055	IC	Educare alla cura della stomia	teaching about stoma care	410091003	Ostomy care education (procedure)
114	10033086	IC	Educare il caregiver	teaching caregiver	460617003	Education of caregiver (situation)
115	10033119	IC	Educare la famiglia a seguire comportamenti salutari	teaching family about health seeking behaviour	710320006	Family education about health seeking behavior (situation)
116	10033161	IC	Trattamento della tracheostomia	tracheostomy care	385858000	Tracheostomy care (regime/therapy)
117	10033188	IC	Trasferire il paziente	transferring patient	107724000	Patient transfer (procedure)
118	10033208	IC	Cura delle lesioni traumatiche	traumatic wound care	225358003	Wound care (regime/therapy)
119	10033254	IC	Trattamento delle ulcere	ulcer care	440461005	Skin ulcer care (regime/therapy)
120	10033277	IC	Cura del catetere urinario	Urinary Catheter Care	429723008	Procedure involving urinary catheter (procedure)
121	10033323	IC	Pesare il paziente	weighing patient	39857003	Weighing patient (procedure)
122	10033347	IC	Cura delle lesioni	wound care	225358003	Wound care (regime/therapy)
123	10033368	IC	Valutare i bisogni	assessing needs	225343006	Assessment of needs (procedure)
124	10033876	IC	Valutare le conoscenze del caregiver	assessing caregiver knowledge	710840008	Assessment of caregiver knowledge (situation)
125	10033905	IC	Valutare il rischio di ipertermia	assessing risk for hyperthermia	709512009	Assessment of risk for hyperthermia (procedure)
126	10033922	IC	Valutare l'integrità cutanea	assessing skin integrity	711041003	Assessment of skin integrity (procedure)
127	10033933	IC	Valutare l'integrità cutanea prima dell'intervento chirurgico	assessing skin integrity before operation	710556008	Pre-operative assessment of skin integrity (procedure)
128	10034048	IC	Valutare il rischio di infezioni dopo un'operazione	evaluating risk for infection after operation	711126000	Post-surgical assessment for risk of infection (procedure)
129	10034053	IC	Valutare la risposta alla gestione del dolore	evaluating response to pain management	370810001	Evaluation of response to pain management interventions (procedure)

130	10034069	IC	Valutare segni e sintomi di infezioni dopo un'operazione	evaluating signs and symptoms of infection after operation	711127009	Post-surgical monitoring for signs and symptoms of infection
131	10034076	IC	Valutare le vie di infusione e i drenaggi	evaluating tubes and drains	711139001	Evaluation of tubes and drains (procedure)
132	10034200	IC	Inserire un dispositivo di accesso vascolare	inserting vascular access device	429446009	Insertion of vascular catheter (procedure)
133	10034285	IC	Monitorare l'attività cardiaca	monitoring cardiac status	23852006	Cardiac monitoring (regime/therapy)
134	10034961	IC	Educare alla cura delle lesioni	teaching about wound care	15502008	Wound treatment education (procedure)
135	10035152	IC	Fototerapia	light therapy	31394004	Light therapy (procedure)
136	10035168	IC	Allattare il bambino con il biberon	Feeding infant with a bottle	40043006	Bottle feeding of patient (regime/therapy)
137	10035199	IC	Screening del neonato prima della dimissione	screening infant before discharge	710077004	Screening of infant prior to discharge (procedure)
138	10035229	IC	Counselling per l'allattamento al seno	counselling about breastfeeding	711029005	Counseling about breastfeeding (procedure)
139	10035272	IC	Gestire l'ipoglicemia	managing hypoglycaemia	386328006	Hypoglycemia management (procedure)
140	10035286	IC	Gestire l'iperglicemia	managing hyperglycaemia	386326005	Hyperglycemia management (procedure)
141	10035293	IC	Mantenere l'integrità cutanea	maintaining skin integrity	710054004	Maintaining integrity of skin (procedure)
142	10035303	IC	Monitorare il bilancio idrico in entrata	monitoring fluid intake	711001007	Monitoring fluid intake (regime/therapy)
143	10035319	IC	Monitorare il bilancio idrico in uscita	monitoring fluid output	711000008	Monitoring fluid output (regime/therapy)
144	10035326	IC	Monitorare lo stato neurologico	monitoring neurological status	182793009	Neurological monitoring regime (regime/therapy)
145	10035335	IC	Monitorare la perfusione tissutale	monitoring tissue perfusion	710988007	Monitoring of tissue perfusion (regime/therapy)
146	10035342	IC	Promuovere l'attaccamento del bambino al caregiver	promoting caregiver child attachment	710141009	Promotion of caregiver child attachment (procedure)
147	10035361	IC	Promuovere la marsupioterapia	promoting skin to skin technique	386342003	Kangaroo care (regime/therapy)
148	10035433	IC	Valutare la sindrome da astinenza	assessing withdrawal	711008001	Assessment of substance withdrawal (procedure)
149	10035451	IC	Misurare la circonferenza cranica	measuring head circumference	56792006	Measurement of skull circumference (procedure)
150	10035678	IC	Valutare la dimissibilità	assessing readiness for discharge	711051002	Assessment of readiness for discharge (procedure)
151	10035887	IC	Collaborare con la famiglia	collaborating with family	711066004	Liaising with family (procedure)
152	10035915	IC	Pianificare l'assistenza	care planning	399684003	Development of care plan (procedure)
153	10036013	IC	Gestire lo stato nutrizionale	managing nutritional status	386372009	Nutrition management (regime/therapy)
154	10036032	IC	Monitorare l'apporto nutrizionale	monitoring nutrition	386374005	Nutritional monitoring (regime/therapy)
155	10036066	IC	Promuovere l'efficace comunicazione della famiglia	promoting effective family communication	710146004	Promotion of effective family communication (procedure)

156	10036078	IC	Promuovere il sostegno familiare	promoting family support	710149006	Promotion of family support (procedure)
157	10036218	IC	Promuovere il ruolo del caregiver	promoting caregiver role	710142002	Promotion of caregiver role (procedure)
158	10036315	IC	Attuare il programma di isolamento	implementing seclusion regime	90278001	Secluding patient (procedure)
159	10036343	IC	Monitorare la sindrome da astinenza	monitoring withdrawal	710986006	Monitoring drug withdrawal (regime/therapy)
160	10036475	IC	Valutare la funzionalità intestinale	assessing bowel status	268389004	Bowel assessment (procedure)
161	10036499	IC	Valutare la funzionalità urinaria	assessing urinary status	711011000	Assessment of urinary status (procedure)
162	10036554	IC	Valutare la risposta all'anestesia dopo l'intervento	evaluating response to anaesthesia after operation	49730009	Post-operative follow-up, anesthesia (procedure)
163	10036577	IC	Mantenere l'accesso venoso	maintaining intravenous access	386493006	Venous access device maintenance (procedure)
164	10036667	IC	Sospendere la terapia endovena	discontinuing intravenous therapy	225203003	Removing intravenous infusion (procedure)
165	10036680	IC	Rimuovere il catetere vescicale	discontinuing urinary catheter	286738000	Removal of urinary system catheter (procedure)
166	10036717	IC	Favorire un'efficace eliminazione intestinale	promoting effective bowel elimination	710145000	Promotion of effective bowel elimination (procedure)
167	10036740	IC	Valutare il rischio di apnea	assessing risk for apnoea	711053004	Assessment of risk for apnea (procedure)
168	10036755	IC	Valutare l'allattamento al seno	assessing breastfeeding	709261005	Assessment of breastfeeding (procedure)
169	10036764	IC	Valutare il sonno	assessing sleep	445221009	Assessment of sleep pattern (procedure)
170	10036772	IC	Valutare lo stato neurologico	assessing neurological status	225398001	Neurological assessment (procedure)
171	10036786	IC	Valutare la funzionalità respiratoria	assessing respiratory status	422834003	Respiratory assessment (procedure)
172	10036819	IC	Misurare la circonferenza toracica	measuring chest circumference	711007006	Measurement of chest circumference (procedure)
173	10036826	IC	Misurare la frequenza cardiaca	measuring heart rate	4625008	Apical pulse taking (procedure)
174	10036857	IC	Insegnare ai familiari l'impiego dei dispositivi	teaching family about device	710745006	Family education about device (situation)
175	10036874	IC	Spiegare ai familiari i test diagnostici	teaching family about diagnostic test	710746007	Family education about diagnostic test (situation)
176	10036916	IC	Prevenire le infezione	preventing infection	386335003	Infection protection (procedure)<br>En Preventing infection
177	10036928	IC	Insegnare ai familiari la prevenzione delle infezioni	teaching family about preventing infection	709272000	Family education about infection prevention (situation)
178	10036971	IC	Insegnare ai familiari a monitorare la funzionalità respiratoria	teaching family about monitoring respiratory status	709275003	Family education about monitoring respiratory status (situation)

179	10036992	IC	Monitorare il livello di bilirubina	monitoring bilirubin level	302787001	Bilirubin measurement (procedure)
180	10037037	IC	Somministrare un integratore alimentare	administering nutritional supplement	709542007	Administration of nutritional supplement (procedure)
181	10037044	IC	Somministrare vitamine	administering vitamin	709543002	Administration of vitamin (procedure)
182	10037071	IC	Valutare l'età gestazionale	assessing gestational age	709479007	Assessment of fetal gestational age (procedure)
183	10037092	IC	Monitorare la terapia respiratoria	monitoring respiratory therapy	425123005	Respiratory therapy surveillance (regime/therapy)
184	10037102	IC	Assistere il neonato	infant care	386333005	Infant care (regime/therapy)
185	10037211	IC	Monitorare la motilità intestinale	monitoring bowel motility	711003005	Monitoring bowel motility (regime/therapy)
186	10037248	IC	Somministrare un antipiretico	administering antipyretic	709541000	Administration of antipyretic (procedure)
187	10037351	IC	Mantenere la pervietà delle vie aeree	maintaining airway clearance	710971000	Maintaining clear airway (procedure)
188	10037398	IC	Mantenere l'isolamento	maintaining isolation technique	40174006	Isolation procedure (procedure)
189	10037490	IC	Preparare la salma	post mortem care	133904006	Postmortem care (regime/therapy)
190	10037881	IC	Valutare il bilancio idrico	assessing fluid balance	710853006	Assessment of fluid balance (procedure)
191	10038131	IC	Insegnare alla famiglia come eseguire l'igiene	teaching family about hygiene pattern	710753003	Family education about hygiene (situation)
192	10038385	IC	Indirizzare ai servizi territoriali	referring to community service	710915002	Referral to community service (procedure)
193	10038550	IC	Insegnare ai familiari le tecniche di trasferimento/mobilizzazione	teaching family about transfer technique	709270008	Family education about transfer technique (situation)
194	10038718	IC	Gestire i sintomi della sindrome da astinenza	managing withdrawal symptom	710059009	Management of withdrawal symptom (procedure)
195	10038741	IC	Programmare un appuntamento di follow up	scheduling follow up appointment	1156892006	Scheduling of follow up appointment (procedure)
196	10038929	IC	Monitorare il dolore	monitoring pain	710995003	Monitoring pain (regime/therapy)
197	10039221	IC	Limitare le stimolazioni	minimising stimulation	710978006	Minimizing environmental stimuli (procedure)
198	10039245	IC	Misurare le entrate di liquidi	measuring fluid intake	711006002	Measurement of fluid intake (procedure)
199	10039250	IC	Misurare le uscite di liquidi	measuring fluid output	711005003	Measurement of fluid output (procedure)
200	10039311	IC	Somministrare una trasfusione di sangue	blood therapy	710953007	Management of administration of blood product (procedure)
201	10039324	IC	Somministrare la terapia elettrolitica	electrolyte therapy	47451004	Administration of electrolytes (procedure)

202	10039330	IC	Somministrare i liquidi	fluid therapy	103744005	Administration of intravenous fluids (procedure)
203	10039369	IC	Somministrare Ossigeno terapia	oxygen therapy	57485005	Oxygen therapy (procedure)
204	10039395	IC	Valutare l'allattamento al seno post partum	postpartum breastfeeding assessment	710345009	Assessment of postpartum breastfeeding (procedure)
205	10039416	IC	Collaborare con il team multidisciplinare	collaborating with interprofessional team	225971008	Liaising with multidisciplinary team (procedure)
206	10039428	IC	Iniziare l'allattamento al seno	initiating breastfeeding	431868002	Initiation of breastfeeding (regime/therapy)
207	10039437	IC	Promuovere l'allattamento al seno	promoting exclusive breastfeeding	710123001	Promotion of exclusive breastfeeding (procedure)
208	10039492	IC	Indirizzare ai gruppi di sostegno per l'allattamento al seno	referring to breastfeeding support group	431340005	Referral to breast feeding peer support service (procedure)
209	10039542	IC	Promuovere l'allattamento	advocating for breastfeeding	710952002	Advocating for breastfeeding (procedure)
210	10039561	IC	Valutare l'allattamento al seno	evaluating breastfeeding	711082003	Assessment of breastfeeding behavior (procedure)
211	10039574	IC	Collaborare con la paziente per il piano di allattamento al seno	collaborating with patient on breastfeeding plan	712554001	Liaising with patient on breastfeeding plan (procedure)
212	10039601	IC	Collaborazione con lo specialista della terapia del dolore	collaborating with pain specialist	709756007	Liaising with pain management specialist (procedure)
213	10039705	IC	Attuare il Comfort Care	implementing comfort care	133918004	Comfort measures (regime/therapy)
214	10039751	IC	Valutare la sicurezza ambientale	assessing environmental safety	225341008	Environmental safety assessment (procedure)
215	10039767	IC	Valutare le conoscenze relative alla sicurezza ambientale	assessing knowledge of environmental safety	709484001	Assessment of knowledge of environmental safety (procedure)
216	10039780	IC	Valutare le conoscenze relative alla prevenzione delle cadute	assessing knowledge of fall prevention	709485000	Assessment of knowledge of fall prevention (procedure)
217	10039808	IC	Avviare il controllo infermieristico dell'analgesia	initiating nurse controlled analgesia	710222001	Initiation of nurse controlled analgesia (procedure)
218	10040211	IC	Prevenire le cadute	fall prevention	386296001	Fall prevention (procedure)
219	10040224	IC	Prevenire la formazione di lesioni da pressione	pressure ulcer prevention	225996006	Pressure ulcer prevention (procedure)
220	10040269	IC	Istruire la famiglia riguardo alla prevenzione delle cadute	teaching family about fall prevention	710754009	Family education about fall prevention (situation)
221	10040435	IC	Collaborare con il nutrizionista	collaborating with nutritionist	709763007	Liaising with nutritionist (procedure)
222	10040461	IC	Valutare il rischio di complicanze della stomia	assessing risk for stoma complication	709514005	Assessment of risk for stoma complication (procedure)

223	10040529	IC	Valutare la stomia	assessing stoma	225192007	Stoma assessment (regime/therapy)
224	10040538	IC	Valutare la cute peristomale	assessing peristomal skin	709497004	Assessment of peristomal skin (procedure)
225	10040604	IC	Insegnare le complicanze della stomia	teaching about stoma complications	710892004	Education about stoma complications (procedure)
226	10040708	IC	Preparare la terapia farmacologica	medication handling	710977001	Safe storage and management of medication (procedure)
227	10040712	IC	Insegnare a preparare la terapia farmacologica	teaching about medication handling	698530005	Education about safe storage and management of medication (procedure)
228	10040847	IC	Valutare le lesioni da pressione	assessing pressure ulcer	225393005	Pressure ulcer assessment (procedure)
229	10040852	IC	Monitorare il bilancio idrico	monitoring fluid balance	430147008	Fluid balance monitoring (regime/therapy)
230	10040932	IC	Valutare il rischio di disidratazione	assessing risk for dehydration	710567009	Assessment of risk for dehydration (procedure)
231	10041126	IC	Valutazione della cute	skin assessment	225397006	Skin assessment (procedure)
232	10041135	IC	Uso di tecniche di mobilizzazione	use transfer technique	710960001	Moving patient using transfer technique (procedure)
233	10041212	IC	Misurare la glicemia	measuring blood glucose	33747003	Glucose measurement, blood (procedure)
234	10041254	IC	Sostenere il morire con dignità	supporting dignified dying	710819007	Support for dignified dying (regime/therapy)
235	10041721	IC	Gestire la febbre	managing fever	386305005	Treatment of fever (procedure)
236	10041732	IC	Insegnare ai familiari come prevenire le infezioni crociate	teaching family about preventing cross infection	709274004	Family education about prevention of cross infection (situation)
237	10041784	IC	Usare tecniche asettiche	use aseptic technique	370822003	Implementation of aseptic technique (procedure)
238	10042507	IC	Gestire la sicurezza ambientale	environmental safety management	385873004	Environmental safety management (procedure)
239	10042856	IC	Valutare lo stato di perfusione tissutale periferica	assessing peripheral tissue perfusion	713135003	Assessment of peripheral tissue perfusion (procedure)
240	10042873	IC	Valutare la funzione neurovascolare periferica	assessing peripheral neurovascular function	713134004	Assessment of peripheral neurovascular function (procedure)
241	10042936	IC	Monitorare la guarigione delle lesioni	monitoring wound healing	372032001	Evaluation of wound healing progress (procedure)
242	10042943	IC	Valutare le lesioni	assessing injury	225391007	Injury assessment (procedure)
243	10042962	IC	Effettuare la Rianimazione cardiopolmona-re	cardiopulmona-ry resuscitation	89666000	Cardiopulmonary resuscitation (procedure)
244	10043464	IC	Cura della frattura	Fracture Care	385691007	Fracture care (regime/therapy)
245	10043602	IC	Disostruire	performing faecal disimpaction	313280002)	Manual evacuation of feces from rectum (procedure)

246	10043618	IC	Eseguire un clistere	performing enema	61919008	Giving patient an enema (procedure)
247	10043639	IC	Spiegare come effettuare un clistere	teaching about giving an enema	385710001	Enema administration education (procedure)
248	10043656	IC	Valutare la diarrea	assessing diarrhoea	408873001	Diarrhea care assessment (procedure)
249	10043884	IC	Monitorare gli effetti collaterali della terapia farmacologica	monitoring medication side effect	410397006	Medication action/side effects surveillance (regime/therapy)
250	10043995	IC	Collaborare con una equipe multiprofessionale per la cura delle lesioni	collaborating with interprofessional team on wound care	1155756009	Liaising with multidisciplinary team on wound care (procedure)
251	10044021	IC	Valutare le lesioni da agenti chimici	assessing chemical injury	370793007	Evaluation for signs and symptoms of chemical injury (procedure)
252	10044078	IC	Effettuare il massaggio Cardiaco esterno	chest compressions	83099006	Manual external cardiac massage with closed chest (procedure)
253	10044097	IC	Effettuare la Rianimazione polmonare	pulmonary resuscitation	33050008	Pulmonary resuscitation (procedure)
254	10044176	IC	Valutare l'apporto di liquidi	assessing fluid intake	1153454009	Assessment of fluid intake (procedure)
255	10044182	IC	Valutare segni e sintomi di infezione	assessing signs and symptoms of infection	1153455005	Assessment of signs and symptoms of infection (procedure)
256	10044195	IC	Valutare la risposta al trattamento	evaluating response to treatment	225953001	Evaluating response to treatment (procedure)
257	10044512	IC	Gestire la sedazione	managing sedation	406187008	Sedation management (regime/therapy)
258	10044633	IC	Raccogliere campioni di sangue venoso	collecting venous blood specimen	28520004	Venipuncture for blood test (procedure)
259	10044679	IC	Curare la sonda enterale	Enteral Tube Care	384745002	Maintenance of gastrointestinal tract tube (procedure)
260	10044729	IC	Trattare la stipsi	treating constipation	389082000	Constipation care (regime/therapy)
261	10044738	IC	Insegnare a misurare la temperatura corporea	teaching about measuring body temperature	410192007	Temperature taking education (procedure)
262	10044819	IC	Terapia inalatoria	inhalation therapy	1366004	Inhalation therapy procedure (procedure)
263	10044835	IC	Insegnare come effettuare la terapia inalatoria	teaching about inhalation therapy	385855002	Inhalation therapy education (procedure)
264	10044888	IC	Insegnare la cura della tracheostomia	teaching about tracheostomy care	410212001	Tracheostomy care education (procedure)
265	10044890	IC	Aspirare le vie aeree	suctioning the airway	230040009	Airway suction technique (procedure)
266	10044992	IC	Uso di Tecniche di rilassamento	use relaxation technique	64299003	Relaxation training therapy (regime/therapy)
267	10045131	IC	Cambiare la medicazione di una ferita	wound dressing change	18949003	Change of dressing (procedure)

268	10045149	IC	Insegnare come cambiare una medicazione	teaching about wound dressing change	385948000	Dressing change education (procedure)
269	10045177	IC	Valutare l'edema	assessing oedema	385931008	Edema control assessment (procedure)
270	10045274	IC	Curare l'urostomia	Urostomy Care	50049003	Urostomy management and care (regime/therapy)
271	10045411	IC	Insegnare le tecniche di alimentazione	teaching about feeding technique	385817007	Patient feeding technique education (procedure)
272	10045815	IC	Somministrare la terapia intradermica	administering intracutaneous medication	386355002	Administration of drug or medicament via intradermal route (procedure)
273	10045827	IC	Somministrare la terapia intramuscolare	administering intramuscular medication	386356001	Administration of drug or medicament via intramuscular route (procedure)
274	10045836	IC	Somministrare la terapia endovenosa	administering intravenous medication	386358000	Administration of drug or medicament via intravenous route (procedure)
275	10045843	IC	Somministrare la terapia sottocutanea	administering subcutaneous medication	386362006	Administration of drug or medicament via subcutaneous route (procedure)
276	10045940	IC	Valutare il rischio di Interazioni farmacologiche avverse	assessing risk for adverse medication interaction	713139009	Assessment of risk of adverse drug interaction (procedure)
277	10046052	IC	Raccogliere campioni di sangue arterioso	collecting arterial blood specimen	713143008	Collection of arterial blood specimen (procedure)
278	10046068	IC	Raccogliere campioni di sangue capillare	collecting capillary blood specimen	1048003	Capillary specimen collection (procedure)
279	10046075	IC	Curare la colostomia	colostomy care	717252006	Colostomy care (regime/therapy)
280	10046099	IC	Mediazione culturale	cultural brokerage	386252001	Culture brokerage (procedure)
281	10046113	IC	Curare il tubo di drenaggio	Drainage Tube Care	385944003	Maintenance of drainage tube (procedure)
282	10046145	IC	Cura della sonda gastrica	Gastric Tube Care	385809004	Gastrostomy/nasogastric tube care management (procedure)
283	10046150	IC	Nutrire il paziente	feeding patient	75118006	Feeding patient (regime/therapy)
284	10046178	IC	Attuare la nutrizione enterale	Implementing Enteral Feeding	229912004	Enteral feeding (regime/therapy)
285	10046184	IC	Attuare la nutrizione parenterale	implementing parenteral feeding	25156005	Intravenous feeding of patient (regime/therapy)
286	10046412	IC	Cura delle lesioni aperte	care of open wound	1153457002	Care of open wound (regime/therapy)
287	10046477	IC	Fisioterapia respiratoria, drenaggio posturale	chest physiotherapy	34431008	34431008 Physiotherapy of chest (regime/therapy)
288	10046579	IC	Somministrare la terapia inalatoria	administering inhalent medication	243132000	Inhaled drug administration (procedure)

289	10046790	IC	Valutare il comportamento	assessing behaviour	225385005	Behavioral assessment (procedure)
290	10046816	IC	Valutare il comportamento del caregiver	assessing caregiver behaviours	733853001	Assessment of caregiver behavior (situation)
291	10046885	IC	Valutare quanto la famiglia è pronta per il Self management	assessing family readiness for self management	733866001	Assessment of family readiness for self-management (situation)
292	10050140	IC	Valutare l'udito	assessing hearing	427247008	Hearing assessment (procedure)
293	10050155	IC	Valutare la deglutizione	assessing swallowing	440363007	Evaluation of oral and pharyngeal swallowing function (procedure)
294	10050164	IC	Valutare le urine	assessing urine	167217005	Urine examination (procedure)
295	10050172	IC	Valutare le feci	assessing faeces	167592004	Examination of feces (procedure)
296	10050186	IC	Valutare la coscienza, lo stato di coscienza	assessing consciousness	444714004	Assessment of consciousness level (procedure)
297	10050206	IC	Valutare l'espettorato	assessing sputum	269911007	Sputum examination (procedure)
298	10050223	IC	Valutare i movimenti corporei	assessing body movement	733919004	Assessment of body movement (procedure)
299	10050234	IC	Valutare la necrosi	assessing necrosis	733871008	Assessment of necrosis of skin (procedure)
300	10050332	IC	Cambiare il pannolino	changing diaper	733923007	Change of diaper (procedure)
301	10050350	IC	Applicare una pomata, un unguento, una crema, un gel	applying ointment	733925000	Application of ointment (procedure)
302	10050378	IC	Collaborare con il fisioterapista	collaborating with physiotherapist	115575800 5	Liaising with physiotherapist (procedure)
303	10050384	IC	Diminuire il rumore	decreasing noise	733849003	Provision of quiet environment (procedure)
304	10050537	IC	Somministrare la terapia per via rettale	administering rectal medication	386360003	Administration of drug or medicament via rectal route (procedure)
305	10050544	IC	Valutare le misure di sicurezza	assessing safety measure	385869002	Safety precautions assessment (procedure)
306	10050592	IC	Auscultare i polmoni	auscultating lung	449264008	Auscultation of lower respiratory tract (procedure)
307	10050626	IC	Pulire la mucosa orale	cleaning oral mucous membrane	115345900 4	Cleaning of oral mucous membrane (procedure)
308	10050657	IC	Attuare lo svezzamento ventilazione	implementing ventilatory weaning	243174005	Weaning from mechanically assisted ventilation (procedure)
309	10050688	IC	Gestire un sanguinamento arterioso	managing arterial blood flow		
310	10050690	IC	Gestire il sanguinamento	managing bleeding	51241000	Control of hemorrhage (procedure)
311	10050724	IC	Gestire lo shock cardiogeno	managing cardiogenic shock	386433009	Shock management: cardiac (procedure)

312	10050730	IC	Gestire un drenaggio toracico	managing chest tube	715592005	Chest drain care management (procedure)
313	10050769	IC	Gestire il dispositivo per l'alimentazione	managing feeding device		
314	10050795	IC	Gestire lo shock ipovolemico	managing hypovolemic shock	386435002	Shock management: compromised volume (procedure)
315	10050851	IC	Gestire un dispositivo per la respirazione	managing respiratory device	121733408	Management of respiratory device (procedure)
316	10050867	IC	Gestire lo shock	managing shock	386432004	Shock management (procedure)
317	10050920	IC	Promuovere uno stato nutrizionale adeguato	promoting positive nutritional status	115695800 7	Promotion of food and nutrient intake to support target weight and body
318	10050949	IC	Promuovere il sonno	promoting sleep	117258304	Promotion of sleep hygiene (procedure)
319	10051156	IC	Facilitare il riposo	facilitating rest	115668500 3	Facilitation of rest (procedure)
320	10051194	IC	Facilitare un sonno efficace	facilitating positive sleep	115668700 6	Facilitation of adequate sleep (procedure)
321	10051248	IC	Esaminare l'addome	examining abdomen	225162003	Examination of abdomen (procedure)
322	10051586	IC	Terapia Respiratoria	respiratory therapy	53950000	Respiratory therapy (procedure)
323	10051627	IC	Stimolare il neonato	stimulating newborn	115583400 4	Stimulation of newborn (procedure)
324	10051643	IC	Monitorare l'equilibrio acido base	monitoring acid base balance	386506007	Acid-base balance monitoring (regime/therapy)
325	10051658	IC	Monitorare l'ascite	monitoring ascites		
326	10051712	IC	Monitorare l'integrità cutanea	monitoring skin integrity	711041003	Assessment of skin integrity (procedure)
327	10051731	IC	Monitorare la risposta allo svezzamento ventilatorio	monitoring response to ventilatory weaning	424139009	Trial for spontaneous breathing (procedure)
328	10051806	IC	Preparare il paziente all'intervento chirurgico	preparing patient for operation	133898004	Preoperative care (regime/therapy)
329	10051913	IC	Indirizzare/rinviare a un terapista della respirazione	referring to respiratory therapy	115576300 9	Referral for respiratory therapy (procedure)
330	10051921	IC	Rimuovere il tubo endotracheale	removing endo tracheal tube	271280005	Removal of endotracheal tube (procedure)
331	10051966	IC	Aspirare il tubo endotracheale	suctioning endo tracheal tube	225715000	Endotracheal tube suction (procedure)
332	10051978	IC	Aspirare la cavità orale	suctioning oral cavity	115583500 3	Suction of oral cavity (procedure)
