# Supplementary material for: Developing a Subset of ICNP® Terminology for NICU and Neonatology Settings
Source: Healthcare (Basel). 2026 Feb 27;14(5):594. doi: 10.3390/healthcare14050594 (PMC12984858; doi:10.3390/healthcare14050594)
Supplement: Supplementary file 1 [file healthcare-14-00594-s001.zip › Table S2.pdf]

Table S2. Equivalence table between newly proposed ICNP® terms and SNOMED CT (terms without a match are highlighted in yellow).

	ICNP® Code	Axis	Italian Browser ICNP®	English Browser ICNP®	SCTID	Browser SNOMED CT
1	Unassigned	DC	Addome globoso	abdomen distended	60728008	Swollen abdomen (finding)<br>en Abdomen distended
2	Unassigned	DC	Alterazione dello sviluppo neuro-comportamentale	impaired neuro-behavioral development	700364009	Neurodevelop-mental disorder (disorder)
3	Unassigned	DC	Assenza di addome globoso	no abdominal distenction	2667000<br>+<br>60728008	Absent (qualifier value)<br><br>Swollen abdomen (finding)<br>en Abdomen distended
4	Unassigned	DC	Assenza di dermatite da presidio assorbente	no diaper dermatitis	2667000<br>+<br>91487003	Absent (qualifier value)<br><br>Diaper rash (disorder)<br>en Diaper dermatitis
5	Unassigned	DC	Assenza di lesioni da dispositivo	no medical-device related injuries	1157027004	No pressure injury (situation)
6	Unassigned	DC	Assenza di stravaso	no extravasation	2667000<br>+<br>76676007	Absent (qualifier value)<br><br>Extravasation (morphologic abnormality)
7	Unassigned	DC	Dermatite da presidio assorbente	Diaer dermatitis	91487003	Diaper rash (disorder)<br>en Diaper dermatitis
8	Unassigned	DC	Difficoltà nella coordinazione suzione-deglutizione-respirazione	difficulty coordinating suction - swallow - breath	299758003<br>+<br>299757008	Sucking reflex equivocal (finding)<br><br>Sucking reflex absent (finding)
9	Unassigned	DC	Difficoltà nella suzione	difficulty sucking	288980000	Difficulty sucking (finding)
10	Unassigned	DC	Eccesso di stimoli ambientali	Excessive environmental stimuli	260378005<br>+<br>255710008	Excessive (qualifier value)<br><br>Environmental stimuli (event)
11	Unassigned	DC	Efficace adattamento al respiratore	effective ventilatory adaptation	1338043001	Patient tolerating mechanical ventilation (finding)
12	Unassigned	DC	Efficace coordinazione suzione-deglutizione-respirazione	Effective coordinating suction - swallow - breath	299756004	Sucking reflex present (finding)

13	Unassigned	DC	Efficace suzione	Effective suctioning		
14	Unassigned	DC	Infezione della ferita chirurgica	surgical wound infection	58126003	Postoperative wound infection (disorder)<br>en Infected surgical wound
15	Unassigned	DC	Intolleranza alle procedure	intolerance to procedures	29544009<br><br>+<br>9632001	Intolerance, function (observable entity)<br><br>Nursing procedure (procedure)
16	Unassigned	DC	Lesione da dispositivo	medical-device related injury	709076009<br><br>870354005	Pressure injury caused by device (disorder)<br><br>Neonatal pressure injury of skin (disorder)
17	Unassigned	DC	Mancanza di adattamento al respiratore	Impaired adaptation to ventilator	450450008	Patient ventilator dyssynchrony (finding)
18	Unassigned	DC	Perdita di peso oltre i limiti della norma	weigth loss beyond normal limits	309257005	Excessive weight loss (finding)
19	Unassigned	DC	Rischio di dermatite da presidio assorbente		30207005<br>+<br>91487003	Risk of (contextual qualifier) (qualifier value)<br><br>Diaper rash (disorder)<br>en Diaper dermatitis
20	Unassigned	DC	Rischio di difficoltà nella suzione	risk for difficulty sucking	30207005<br>+<br>288980000	Risk of (contextual qualifier) (qualifier value)<br><br>Difficulty sucking (finding)
21	Unassigned	DC	Rischio di infezione della ferita chirurgica	risk for surgical wound infection	30207005<br>+<br>58126003	Risk of (contextual qualifier) (qualifier value)<br><br>Postoperative wound infection (disorder)<br>en Infected surgical wound
22	Unassigned	DC	Rischio di lesione da dispositivo	risk for medical-device related injury	30207005<br>+<br>709076009	Risk of (contextual qualifier) (qualifier value)<br><br>Pressure injury caused by device (disorder)

23	Unassigned	DC	Rischio di mancanza di adattamento al respiratore	risk for ventilatory impaires adaptation		
24	Unassigned	DC	Rischio di risposta negativa alle tecniche di controllo del dolore non farmacologiche	risk for negative response to non-pharmacological pain management		
25	Unassigned	DC	Rischio di stravaso	risk for extravasation	30207005<br>+<br>76676007	Risk of (contextual qualifier) (qualifier value)<br><br>Extravasation (morphologic abnormality)
26	Unassigned	DC	Risposta negativa alle tecniche controllo del dolore non farmacologiche	negative response to non-pharmacological pain management		
27	Unassigned	DC	Stimoli ambientali controllati	Controlled environmental stimuli	31509003<br>+<br>255710008	Controlled (qualifier value)<br><br>Environmental stimuli (event)
28	Unassigned	DC	Stravaso	extravasation	1142079003	Neonatal extravasation injury (disorder)
29	Unassigned	DC	Sviluppo neurocomportamentale nella norma	effective neuro-behavioral development	860642002	No neurodevelopmental disorder present (situation)
30	Unassigned	DC	tolleranza alle procedure	Tolerating nursing procedures	71412006<br>+<br>9632001	Tolerance, function (observable entity)<br><br>Nursing procedure (procedure)
1	Unassigned	IC	Alimentare il neonato per bocca	Oral feeding	289148009	Normal infant feeding (finding)
2	Unassigned	IC	Alternare la posizione dei dispositivi di monitoraggio	alternating monitoring device position	385974006<br><br>or<br>85874005<br><br>or<br>363108004	Equipment safety management (procedure)<br><br>Equipment safety assessment (procedure)<br><br>Equipment-related management procedure (procedure)
3	Unassigned	IC	Applicare il sacchetto urine	applying urine collection bag	698086002	Collection of urine via pediatric urine collection bag (procedure)

4	Unassigned	IC	Applicare una medicazione compressiva	applying compressive wound dressing	413899004	Compression bandaging (procedure)
5	Unassigned	IC	Aspirare dalla tracheostomia	suctioning via tracheostomy cannula	232703000<br><br>or<br>398083003	Tracheal suction via tracheostomy (procedure)<br><br>Bronchial suction via tracheostomy tube (procedure)
6	Unassigned	IC	Aspirare il retrofaringe	suctioning retropharynx	447153008	Aspiration of pharynx (procedure)
7	Unassigned	IC	Aspirare le secrezioni	suctioning secrections	230040009	Airway suction technique (procedure)
8	Unassigned	IC	Attuare la marsupioterapia	skin to skin care oppure kangaroo care	386342003	Kangaroo care (regime/therapy)
9	Unassigned	IC	Attuare tecniche non farmacologiche per il controllo del dolore	implementing for non-pharmacological strategies for pain management		
10	Unassigned	IC	Coinvolgere nelle cure il genitore, la famiglia	involving parents and family in care	51501005	Hospital admission, parent, for in-hospital child care (procedure)
11	Unassigned	IC	Consolare il neonato	comforting the newborn	386284008<br><br>oppure<br>385895000<br><br>oppure<br>133918004	Environmental management: comfort (regime/therapy)<br><br>Comfort care assessment (procedure)<br><br>Comfort measures (regime/therapy) en Comfort care
12	Unassigned	IC	Attuare la contenzione del neonato con il wrapping	wrapping the newborn	1230128003	Swaddling of baby (regime/therapy)
13	Unassigned	IC	Gestire il livello di luce nell'ambiente	managing ambiental light level	710978006	Minimizing environmental stimuli (procedure) en Measures to limit noise and conversation in the room, keeping light levels low and minimizing tactile stimulation

14	Unassigned	IC	Gestire il livello del rumore nell'ambiente	managing ambiental noise level	710978006	Minimizing environmental stimuli (procedure) en Measures to limit noise and conversation in the room, keeping light levels low and minimizing tactile stimulation
15	Unassigned	IC	Effettuare la stimolazione rettale	rectal stimulation	389082000	Constipation care (regime/therapy)
16	Unassigned	IC	Effettuare i lavaggi nasali	nasal irrigation	69378000	Irrigation of nasal passages (procedure)
17	Unassigned	IC	Effettuare l'emogasanalisi capillare	capillary blood gas analysis	10812009	Blood gases, capillary measurement (procedure)
18	Unassigned	IC	Effettuare il monitoraggio transcutaneo dei gas	Transcoutaneous blood gas monitoring	284028002<br><br>oppure 252465000<br><br>oppure 284035005	Respiratory gas monitoring (regime/therapy)<br><br>Pulse oximetry (procedure) en Transcutaneous pulse oximetry<br><br>Transcutaneous oxygen monitoring (regime/therapy)
19	Unassigned	IC	Effettuare la pronazione del neonato	turning the newborn to a prone position	431182000	Placing subject in prone position (procedure)
20	Unassigned	IC	Fare il bagno preoperatorio al neonato	bathing the newborn before operation	431259009<br><br>or 17315004<br><br>or 8792004	Bathing infant (procedure)<br><br>Bathing patient in incubator (procedure)<br><br>Preoperative preparation of skin (procedure)
21	Unassigned	IC	Gestire il catetere venoso ombelicale	managing umbilical venous catether	386482009 + 1268297002	Maintenance of umbilical catheter (procedure) en Umbilical line tube care<br><br>umbilical venous catheter (physical object)

22	Unassigned	IC	Gestire il catetere arterioso ombelicale	managing umbilical arterial catether	386482009<br>+<br>1268296006	Maintenance of umbilical catheter (procedure)<br>en Umbilical line tube care<br><br>Umbilical arterial catheter (physical object)
23	Unassigned	IC	Gestire il dispositivo di monitoraggio dell'attività cerebrale del neonato	managing brain-activity monitoring device	133909001<br>+<br>252737003	Maintenance of device (procedure)<br><br>Continuous monitoring using cerebral function analysing monitor (procedure)
24	Unassigned	IC	Gestire il tubo endotracheale	managing endotracheal tube	409088002	Tube care: endotracheal (procedure)
25	Unassigned	IC	Gestire la derivazione ventricolare esterna	managing external ventricular drain	386484005<br><br><br>oppure<br>230869001<br><br><br><br>oppure<br>153008441000119109	Ventriculostomy/ lumbar drain care (procedure)<br><br>External drainage procedure from ventricle of brain (procedure)<br><br>Intracranial ventricular fluid external drainage catheter in situ (finding)
26	Unassigned	IC	Gestire lo stravaso	managing extravasation	1142079003<br>+<br>711107008	Neonatal extravasation injury (disorder)<br><br>Injury care (regime/therapy)
27	Unassigned	IC	Gestire un catetere venoso centrale	Managing a central line	226005007	Care of central line (procedure)
28	Unassigned	IC	Gestire un accesso venoso periferico	Managing a periferical venous line	386493006	Venous access device maintenance (procedure)
29	Unassigned	IC	Indurre l'ipotermia terapeutica	inducing terapeuthical hypothermia	308693008<br><br><br>or<br>1713004	Induction of hypothermia (procedure)<br><br>Induction and maintenance of total body hypothermia (procedure)
30	Unassigned	IC	Insegnare a somministrare il latte con il biberon	teaching about feeding with a bottle	438297004	Education about feeding neonate (procedure)

31	Unassigned	IC	Insegnare come cambiare il pannolino	teaching about changing diaper	362978005<br>+<br>733923007	Medical equipment or device education (procedure)<br><br>Change of diaper (procedure)
32	Unassigned	IC	Insegnare l'uso del tiralatte	teaching about using breast milk pump	362978005<br>+<br>31629005	Medical equipment or device education (procedure)<br><br>Pump extraction of milk from lactating breast (procedure)
33	Unassigned	IC	Mantenere pervie le vie aeree utilizzando la cannula di Mayo	maintaining airways open by Mayo cannula (Guedel cannula) or oropharyngeal cannula	225668003<br><br>or<br>7443007	Insertion of Guedel airway (procedure)<br><br>Insertion of oropharyngeal airway (procedure)
34	Unassigned	IC	Misurare la circonferenza addominale	Measuring abdominal girth	48094003	Abdominal girth measurement (procedure)
35	Unassigned	IC	Misurare la lunghezza	Measuring lenght	14456009	Measuring height of patient (procedure)
36	Unassigned	IC	Monitorare i sintomi di emorragia cerebrale	Monitoring symptoms of cerebral hemorrage	386369002<br>+<br>70611002<br><br>or<br>261808007	Newborn monitoring (regime/therapy)<br><br>Perinatal intraventricular hemorrhage (disorder)<br><br>Neonatal cerebral hemorrhage (disorder)
37	Unassigned	IC	Monitorare il comfort del neonato	monitoring newborn's confort	385897008<br><br>or<br>385895000	Comfort care management (procedure)<br><br>Comfort care assessment (procedure)

38	Unassigned	IC	Monitorare il ristagno gastrico	monitoring gastric residual volume	239516002<br><br>+<br>1162665001<br><br>or<br>310872003	Monitoring procedure (regime/therapy)<br><br>Volume of drainage of gastric contents (observable entity) enThe amount of gastric fluid output from drain.<br><br>Gastric drainage procedure (procedure)
39	Unassigned	IC	Monitorare il vomito	vomit monitoring	442030001<br><br>or<br>167820005<br><br>or<br>364694004	Evaluation of vomitus specimen (procedure)<br><br>Vomit examination (procedure)<br><br>Measure of vomit (observable entity)
40	Unassigned	IC	Monitorare la frequenza cardiaca	heart rate monitoring	47101004	Cardiotachometry (regime/ therapy)<br>en Heart rate monitoring
41	Unassigned	IC	Monitorare la frequenza respiratoria	respiratory rate monitoring	284017001	Respiratory rate monitoring (regime/therapy)
42	Unassigned	IC	Monitorare la pervietà dell'alvo	monitoring alvo oppure<br>bowel patency	699040006<br><br>or<br>249521002	Stool monitoring (regime/therapy)<br><br>Frequency of bowel action (observable entity)
43	Unassigned	IC	Monitorare la pressione arteriosa cruenta	monitoring invasive arterial blood pressure	77938009	Arterial pressure monitoring, invasive method (regime/therapy)
44	Unassigned	IC	Monitorare la sedazione tramite strumento di valutazione	monitoring sedation by an evaluation tool	445536008<br><br>or<br>1284907001	Assessment using assessment scale (procedure)<br><br>Assessment using Neonatal Pain Assessment and Sedation Scale (procedure)

45	Unassigned	IC	Monitorare la suzione	monitoring suction	230126006<br><br>or<br>289145007<br><br>or<br>1172954007<br><br>or<br>364809004<br><br>or<br>364786002	Finding relating to infant feeding (finding)<br>en Baby feeding observation<br><br>Finding of infant feeding pattern (finding)<br>en Observation of infant feeding pattern<br><br>Evaluation of infant formula feeding (procedure)<br><br>Finding related to ability to latch on to breast for feeding (finding)<br><br>Finding of eating ability (finding)
46	Unassigned	IC	Monitorare la tolleranza alimentare	monitoring food tolerance	276716007	Infant not tolerating feeds (finding)
47	Unassigned	IC	Monitorare la presenza di ernie inguinali	monitoring ingunal hernia	170208008<br><br>or<br>284426008	Child examination: herniae (procedure)<br><br>Examination of hernial orifices (procedure)
48	Unassigned	IC	Monitorare le secrezioni	secretions monitoring		
49	Unassigned	IC	Monitorare l'idrocefalo	monitoring hydrocephalus	56792006<br><br>or<br>363811000<br><br>or<br>170190000	Measurement of skull circumference (procedure)<br><br>Head circumference measure (observable entity)<br><br>Child examination: fontanel (procedure)
50	Unassigned	IC	Posizionare un accesso venoso periferico	positioning periferal venous line	392230005<br><br>or<br>735414004	Catheterization of vein (procedure)<br><br>Insertion of catheter into peripheral vein (procedure)
51	Unassigned	IC	Posizionare un catetere arterioso ombelicale	positioning umbilical arterial catether	233519002	Umbilical artery cannula insertion (procedure)

52	Unassigned	IC	Posizionare un catetere venoso centrale		233527006	Central venous cannula insertion (procedure)
53	Unassigned	IC	Posizionare un catetere venoso ombelicale	positioning umbiilical venous catether	42550007	Catheterization of umbilical vein (procedure)
54	Unassigned	IC	Posizionare una sonda gastrica	positioning gastric tube	87750000	Insertion of nasogastric tube (procedure)
55	Unassigned	IC	Prevenire la dermatite da presidio assorbente	preventing diaper rash	169443000<br>+<br>91487003	Preventive procedure (procedure)<br><br>Diaper rash (disorder)
56	Unassigned	IC	Regolare<br>(la termoculla)<br>Azione ICNP®	Regulating	438298009	Control of infant incubator temperature (procedure)
57	Unassigned	IC	Somministrare il colostro	administering colostrum	75118006<br>+<br>53875002	Feeding patient (regime/therapy)<br><br>Colostrum (substance)
58	Unassigned	IC	Somministrare emoderivati	administering blood products	116762002	Administration of blood product (procedure)
59	Unassigned	IC	Somministrare il latte artificiale	administering formula milk	75118006<br>+<br>443231000124101<br><br>or<br>40043006	Feeding patient (regime/therapy)<br><br>Premature infant formula (product)<br><br>Bottle feeding of patient (regime/therapy)
60	Unassigned	IC	Somministrare il latte con il gavage	administering milk by gavage	229914003	Nasogastric feeding (regime/therapy) en Gastric gavage
61	Unassigned	IC	Somministrare il latte spremuto	administering expressed breast milk	75118006<br>+<br>226790003	Feeding patient (regime/therapy)<br><br>Expressed breast milk (substance)
62	Unassigned	IC	Somministrare ossido nitrico	administering nitric oxide	1255879001	Administration of nitric oxide (procedure)
63	Unassigned	IC	Somministrare surfactante	administering surfactant	434701000124101	Administration of lung surfactant (procedure)
64	Unassigned	IC	Stimolare il neonato in sala parto	stimulating newborn in delivery room	1155834004	Stimulation of newborn (procedure)
65	Unassigned	IC	Stimolare la suzione non nutritiva	stimulating non nutritive suction	386370001	Provision of non-nutritive sucking (regime/therapy)

66	Unassigned	IC	Valutare il grado di autonomia del genitore nella gestione del neonato	evaluate parent's autonomy to care the newborn	1345014007	Parenting skills case management (procedure)
67	Unassigned	IC	Valutare la presenza di meconio	evaluate meconium presence	17695009	Meconium quantitation (procedure)
